# Single-cell genotyping and transcriptomic profiling of mosaic focal cortical dysplasia

**DOI:** 10.1038/s41593-025-01936-z

**Published:** 2025-04-30

**Authors:** Sara Baldassari, Esther Klingler, Lucia Gomez Teijeiro, Marion Doladilhe, Corentin Raoux, Sergi Roig-Puiggros, Sara Bizzotto, Jeanne Couturier, Alice Gilbert, Lina Sami, Théo Ribierre, Eleonora Aronica, Homa Adle-Biassette, Mathilde Chipaux, Denis Jabaudon, Stéphanie Baulac

**Affiliations:** 1 Institut du Cerveau-Paris Brain Institute-ICM, Sorbonne Université, Inserm, CNRS, Hôpital de la Pitié Salpêtrière, Paris, France; 2 VIB-KU Leuven Center for Brain & Disease Research, Leuven, Belgium; 3 KU Leuven Department of Neurosciences, Leuven Brain Institute, Leuven, Belgium; 4 KU Leuven Institute for Single Cell Omics, Leuven, Belgium; 5 Department of Basic Neurosciences, University of Geneva, Geneva, Switzerland; 6 Fondation Campus Biotech Geneva, Geneva, Switzerland; 7 Department of (Neuro)Pathology, Amsterdam UMC, University of Amsterdam, Amsterdam Neuroscience, Amsterdam, The Netherlands; 8 Université Paris Cité, Inserm, NeuroDiderot, Paris, France; 9 Department of Pathology, AP-HP, Lariboisière Hospital, Paris, France; 10 Pediatric Neurosurgery Department, CCMR Epilepsies Rares, European Reference Network EpiCare Member, Rothschild Foundation Hospital, Paris, France; 11 Clinic of Neurology, Geneva University Hospital, Geneva, Switzerland

**Keywords:** Molecular neuroscience, Epilepsy, Neurodevelopmental disorders, Transcriptomics

## Abstract

Focal cortical dysplasia type II (FCDII) is a cortical malformation causing refractory epilepsy. FCDII arises from developmental somatic activating mutations in mTOR pathway genes, leading to focal cortical dyslamination and abnormal cytomegalic cells. Which cell types carry pathogenic mutations and how they affect cell-type-specific transcriptional programs remain unknown. In the present study, we combined several single-nucleus genotyping and transcriptomics approaches with spatial resolution in surgical cortical specimens from patients with genetically mosaic FCDII. Mutations were detected in distinct cell types, including glutamatergic neurons and astrocytes, and a small fraction of mutated cells exhibited cytomegalic features. Moreover, we identified cell-type-specific transcriptional dysregulations in both mutated and nonmutated FCDII cells, including synapse- and neurodevelopment-related pathways, that may account for epilepsy and dysregulation of mitochondrial metabolism pathways in cytomegalic cells. Together, these findings reveal cell-autonomous and non-cell-autonomous features of FCDII that may be leveraged for precision medicine.

## Abstract (web summary)

In this work, the authors performed a single-cell genotyping and transcriptomics analysis, revealing cell-type-specific and nonautonomous effects of mTOR pathway mutations in mosaic focal cortical dysplasia.

## Main

All cells in the body originate from a single dividing fertilized egg, yet daughter cells have different genomes as a result of somatic mosaicism, which in the brain contributes to generating neural cell diversity^1–3^. Moreover, recent studies have demonstrated that brain somatic mutations can cause neurodevelopmental and neuropsychiatric disorders, including the cortical malformation focal cortical dysplasia type II (FCDII)^4,5^. FCDII results in childhood-onset, drug-resistant epilepsy, commonly necessitating neurosurgical resection of the epileptogenic zone, and is the most prevalent cortical malformation in pediatric epilepsy surgery^6^. FCDII lesions can affect small distinct cortical areas or extend to entire hemispheres, as in hemimegalencephaly. FCDII is characterized by the focal disruption of cortical lamination and the presence of characteristic cytomegalic cells, namely, dysmorphic neurons (DNs) and balloon cells (BCs), which are intermingled with cells with normal morphologies in a mosaic pattern^7^. DNs contribute to epileptic discharges and present an enlarged soma with a cytoplasmic accumulation of neurofilaments and Nissl substance^8^. BCs have large cell bodies with opalescent cytoplasm devoid of Nissl substance and are electrically silent. Recently, deep sequencing of surgically resected brain tissue has revealed somatic mutations in the mTOR (mammalian target of rapamycin) pathway genes in up to 60% of FCDII cases^9^. FCDII-causing mutations are either somatic gain-of-function single hits in genes coding for pathway activators (for example, *MTOR*, *PIK3CA*, *AKT3* and *RHEB*) or loss-of-function double-hits (germline and somatic) in genes coding for inhibitors of this pathway (for example, *DEPDC5* and *TSC1*/*-2*)^9–14^. The proportion of mutated cells within the tissue correlates with the size of the lesion^9^. These postzygotic mutations are thought to arise during corticogenesis in dorsal pallium progenitors lining the ventricular zone^15^ and to give rise to cytomegalic cells caused by mTOR hyperactivation, a pathway that regulates cell growth and metabolism^16^. We and others have shown that DNs and BCs carry identical mTOR-activating mutations, suggesting a common origin from a single mutation event during development^12,17^. Yet, the mechanisms by which DNs and BCs contribute to epilepsy, and whether they represent distinct cell types or instead different states of a single-cell type, is mostly unknown. Moreover, a remaining challenge is to address the extent to which seizures reflect an abnormal function of cytomegalic cells and/or non-cell-autonomous effects of mutated cells on neighboring neurons. Although bulk transcriptomic studies of FCDII tissues have been conducted^18–21^, single-cell resolution is essential to resolve the etiopathogenesis of FCDII in which only a few cells are mutated as a result of somatic mosaicism.

To address these questions, we used single-nucleus genotyping and transcriptomic analyses, along with spatial transcriptomics and orthogonal approaches for further validation on ten FCDII surgical cases with somatic mTOR pathway mutations. Our results reveal that DNs and BCs are molecularly distinct from one another, with glutamatergic neuron-like and astrocyte-like identities, respectively, and together represent only a small fraction of all mutated cells in FCDII tissue. We identified cell-type-specific transcriptional programs that are both cell-autonomously and non-cell-autonomously triggered by the mutation, with evidence for dysregulated mitochondrial metabolism specifically in cytomegalic neurons.

## Results

**Fig. 1:**
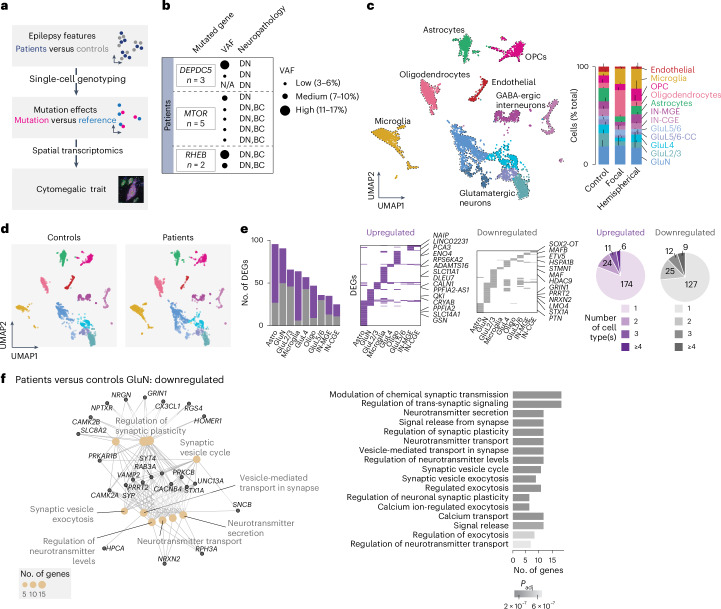
Cell-type-specific dysregulation of synapse and neurodevelopmental pathways in FCDII. **a**, Study workflow. SnRNA-seq was employed to identify transcriptional changes related to epilepsy in FCDII tissues. Genotyping on mutation sites was used to compare Mut. nuclei, which carried the somatic mutation, and Ref. nuclei, where only the reference allele was detected. Integration with spatial resolution linked transcriptional changes to cellular morphology and spatial disorganization. **b**, Patient cohort overview: ten patients with FCDII (pt1–10) and mTOR pathway mutations in *MTOR*, *DEPDC5* (mTOR repressor) and *RHEB* (mTOR activator). Dysplastic areas in pt1, -4 and -9 extended to an entire hemisphere, with pt4 and -9 diagnosed with hemimegalencephaly. N/A, not available (no somatic hit identified). **c**, Left, UMAP of integrated patients and controls, snRNA-seq data with cell-type annotations from a previous study^22^ (Methods). Dashed lines outline cluster densities. Right, cell-type proportions across controls and patients with focal or hemispherical dysplasia. **d**, UMAP visualization showing overlapping cell types between control and patient nuclei, with a subset of GluNs enriched in pt8 and pt9. **e**, Cell-type-specific differential gene expression analysis between focal FCDII and controls. Left, number of DEGs per cell type (significant genes expressed in at least 25% of cells with absolute log_2_(FC) > 0.4). Middle, specific and shared DEGs across cell types; examples of DEGs specific to one cell type are indicated. Right, proportion of DEGs specific to one or more cell types. **f**, Top significant GOs and genes downregulated in FCDII GluNs. CC, corticocortical projection neurons; L, layer.

**Extended Data Fig. 1:**
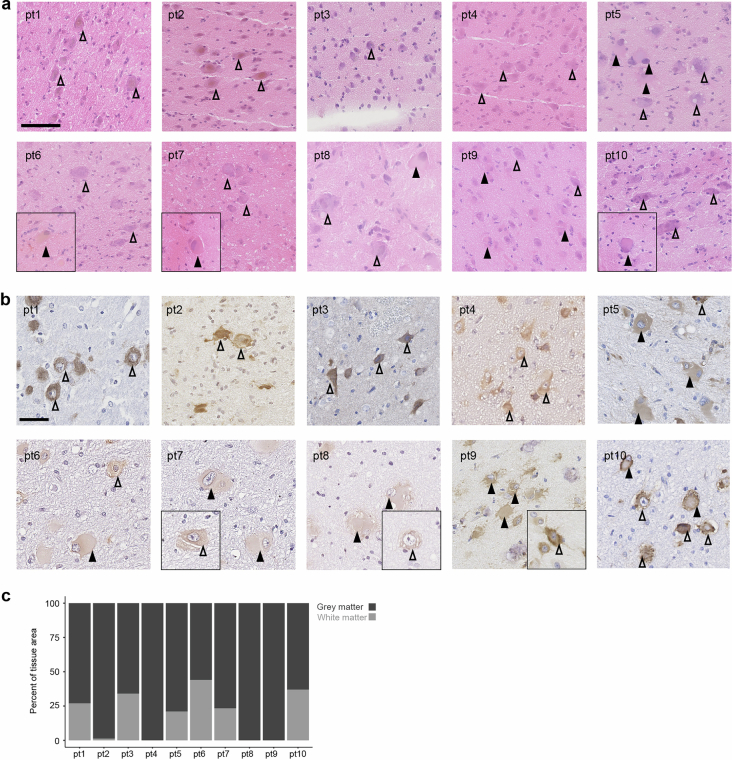
Neuropathological characterization of FCDII brain tissues. **a**, Representative hematoxylin and eosin (HE) staining of 20μm-thick frozen brain sections showing dysmorphic neurons (DN, empty arrowheads) and balloon cells (BC, filled arrowheads) across FCDII patients (n = 1 section/patient). Scale bar: 100μm. Insets for patients pt6, pt7 and pt10 show a representative BC from distinct cortical regions. **b**, Immunohistochemical detection of mTOR pathway activation using phosphorylated S6 (pS6-Ser240/244) antibody on 4μm-thick formalin-fixed paraffin-embedded (FFPE) brain sections (n = 1 section/patient). DN (empty arrowheads) and BC (filled arrowheads) exhibit strong pS6 immunoreactivity. Scale bar: 50μm. Insets for patients pt7-9 show representative DN from distinct cortical regions. **c**, Quantification of grey and white matter proportions in 20μm-thick HE-stained frozen brain sections adjacent to tissue used for single-nucleus RNA sequencing (n = 1 section/patient).

The overall goal of the present study was to investigate cell-type-specific transcriptional dysregulations in a homogeneous collection of FCDII tissues by combining multiple transcriptomic approaches, to characterize epilepsy-associated alterations and mTOR-driven changes, and identify disease-related biomarkers (Fig. 1a). For this purpose, we collected frozen surgical cortical samples from ten children who underwent neurosurgery to treat drug-resistant focal epilepsy, along with three postmortem cortical samples from nonepileptic, age-matched controls (for demographics, and clinical and genetic features, see Supplementary Table 1). The patients had either somatic gain-of-function variants in *MTOR* (*n* = 5) or *RHEB* (*n* = 2) or loss-of-function germline and somatic variants in the mTOR inhibitor *DEPDC5* (*n* = 3). Patients received a neuropathological diagnosis of FCDII, either FCDIIa with only DNs (all *DEPDC5* cases and one *MTOR* case) or FCDIIb with both DNs and BCs (*MTOR* and *RHEB* cases; neuropathology is shown in Extended Data Fig. 1a). The variant allele frequency (VAF, reflecting the percentage of mutated alleles) ranged from 3% in focal lesions to 17% in hemispherical lesions (Fig. 1b). In all patients, DNs and BCs displayed mTOR hyperactivity as shown by phosphorylated S6 (pS6) immunostaining, a readout of mTOR signaling activation (Extended Data Fig. 1b).

### Cell-type-specific transcriptional changes in FCDII cortex

**Extended Data Fig. 2:**
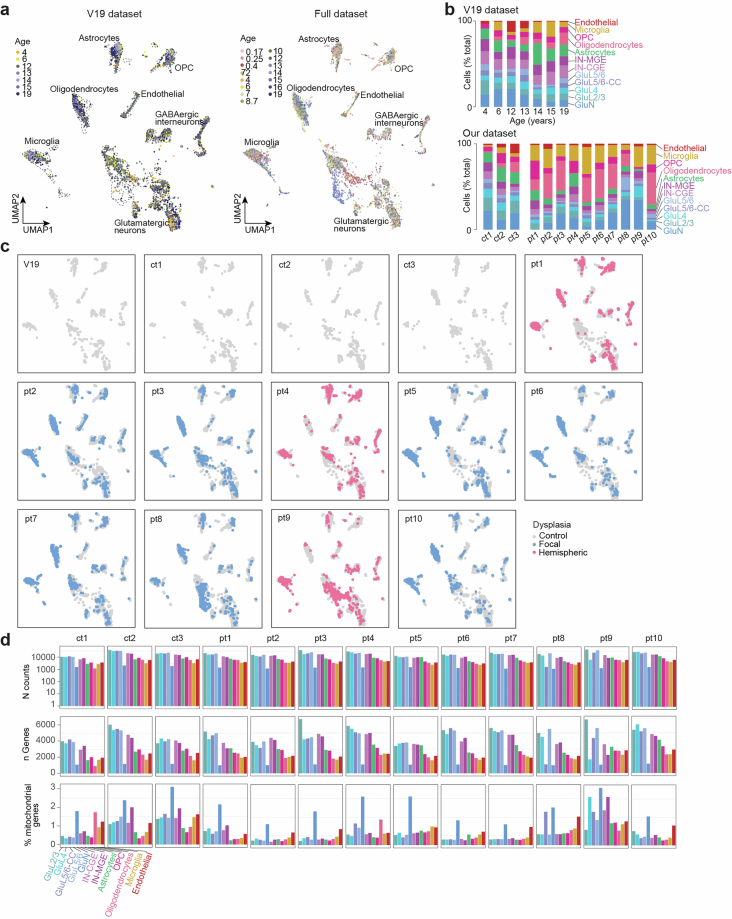
Integration and quality control of single-nucleus RNA-seq (snRNA-seq) data from control and patient brain tissues. **a**, UMAP visualization comparing age distribution of subjects between the reference Velmeshev et al.^22^ postmortem control dataset (V19, left) and the complete integrated dataset from this study (right). Color gradient indicates subject age in years. **b**, Relative proportion of major cell types across individual samples. Cell types include glutamatergic neurons (GluN and GluL2-6), GABAergic interneurons (IN-MGE and IN-CGE), glial cells (astrocytes, oligodendrocytes, OPCs) and other cell types (microglia, endothelial cells). Abbreviations: Glu, glutamatergic; N, neurons; L, layer; CC, cortico-cortical projection neurons; IN-MGE/CGE, interneurons originating from the medial/caudal ganglionic eminence; OPC, oligodendrocyte precursor cells. **c**, Individual UMAP plots showing nucleus distribution for each patient and control. **d**, Quality metrics for snRNA-seq data across cell types and individuals: total count of unique molecular identifiers (UMIs) per nucleus (N counts), mean number of unique genes (N genes) detected per nucleus and percentage (%) of transcripts from mitochondrial genes.

We performed single-nucleus 3′-RNA sequencing (snRNA-seq) of cortical tissues using the 10x Genomics technology, which yielded 178,057 quality-controlled single nuclei from the ten patients with FCDII and the three nonepileptic controls (detailed sample information for each experiment and analysis in Supplementary Table 2). To expand the control dataset, we integrated eight age-matched neurotypical controls from a previous study^22^ and analyzed the distribution of 184,007 nuclei (43,355 control nuclei and 140,652 patient nuclei) according to their transcriptional profile using two-dimensional (2D) uniform manifold approximation and projection (UMAP) dimensionality reduction (Fig. 1c and Extended Data Fig. 2). Based on the expression of canonical brain cell-type markers and *k*-nearest neighbor cell-type predictions built on the control age-matched dataset, we identified seven major cell types: glutamatergic neurons (GluNs) and γ-aminobutyric acid (GABA)-ergic interneurons (derived from medial (IN-MGE) and caudal (IN-CGE) ganglionic eminences), astrocytes, oligodendrocytes, oligodendrocyte precursor cells (OPCs), microglia, as well as endothelial cells (Fig. 1c; snRNA-seq quality metrics are shown in Supplementary Table 3). The relative proportion of these cell types varied between FCDII and control tissues. Notably, microglial cells, oligodendrocytes and OPCs were overrepresented in FCDII samples, possibly reflecting differences in the surgical sampling between controls and patients (postmortem tissue in which the cortical area can be precisely dissected versus postoperative tissue with broader margins of resection extending within the white matter) (Fig. 1c and Extended Data Figs. 1c and 2b).

**Extended Data Fig. 3:**
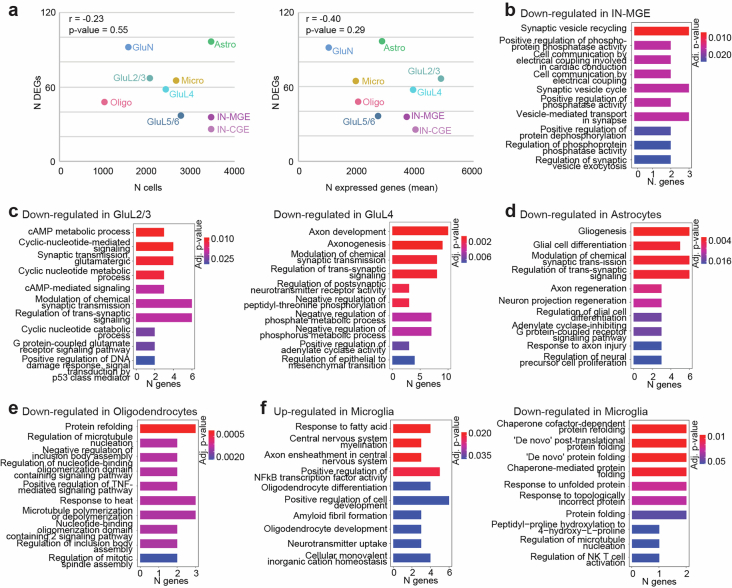
Cell-type-specific DEGs in focal FCDII patients. **a**, Quantitative assessment of differentially expressed genes (DEGs) between patients and controls (log_2_(FC) > 0.4, p-value < 0.05, Fig. 1). Left: Correlation between DEG count and total number of nuclei per cell type. Right: Correlation between DEG count and mean number of genes detected per nucleus in each cell type. Pearson correlation coefficients (R) and p-values are shown. Abbreviations: Astro, astrocytes; Glu, glutamatergic; N, neurons; L, layer; IN-MGE/CGE, interneurons originating from the medial/caudal ganglionic eminence; Micro, microglia; Oligo, oligodendrocytes; OPC, oligodendrocyte precursor cells. **b-f**, Gene Ontology (GO) pathway analysis of DEGs comparing focal FCDII patients to controls for interneurons (**b**), glutamatergic neurons (**c**), astrocytes (**d**), oligodendrocytes (**e**) and microglia (**f**). Top 10 significantly enriched GO terms with adjusted (adj.) p-value < 0.05 are shown per cell type.

Our data show that cell types largely overlapped between patients and controls, as we did not observe new cell clusters common to all patients, although a subset of glutamatergic neurons was enriched in two patients (Fig. 1d and Extended Data Fig. 2c). To examine how molecular pathways are affected in a cell-type-specific manner, we investigated the differentially expressed genes (DEGs) in patients compared with nonepileptic controls for each cell type. We observed broad transcriptional changes, with glutamatergic neurons and astrocytes showing the highest number of DEGs (*n* = 91 DEGs in GluNs, *n* = 96 DEGs in astrocytes), whereas GABA-ergic interneurons were the least affected (*n* = 25 DEGs in IN-CGEs and *n* = 35 DEGs in IN-MGEs) (Fig. 1e and Supplementary Table 4). DEG count differences across cell types were unrelated to cell or gene numbers detected per cell type, supporting genuine biological variation (Extended Data Fig. 3a). Remarkably, most DEGs were unique to individual cell types and gene ontology (GO) enrichment analysis revealed distinct dysregulated pathways across cell types (Fig. 1e and Extended Data Fig. 3b–f), indicating a strong cell-type-specific component to FCDII pathogenesis. For example, in the GluN cluster of patients with FCDII, we observed downregulation of *GRIN1* and *PRRT2*, two genes causing monogenic forms of focal epilepsies, and upregulation of *GLUL*, pointing toward alterations of the glutamate signaling and neurotransmission in glutamatergic neurons. Enriched GO terms in the GluN cluster were mostly associated with neurodevelopment, neurotransmission and synaptic function (for example, *SYT4*, *NPTXR*, *NRXN2* and *STX1A*; Fig. 1f). Together, these results reveal disease-relevant, cell-type-specific molecular dysregulations in the epileptic tissue, probably reflecting and/or contributing to circuit dysfunction and seizure activity.

### Distribution of FCDII somatic mutations across cell types

**Fig. 2:**
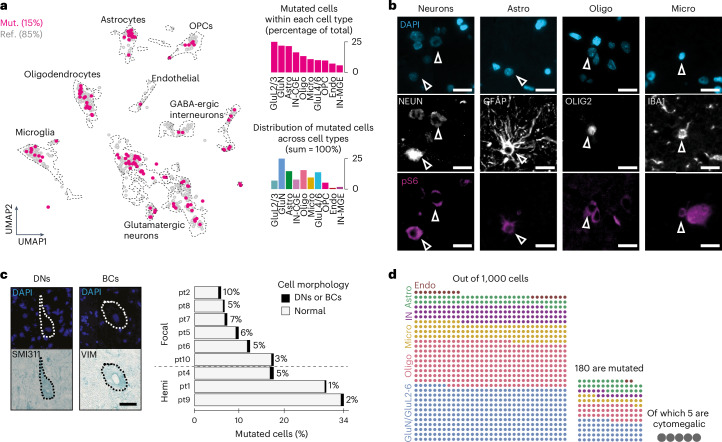
Mutated cells are detected in various cell types and only occasionally exhibit a cytomegalic phenotype. **a**, Distribution of 808 genotyped nuclei in UMAP space: 117 were classified as Mut. (pt10 = 89, pt9 = 25, pt7 = 2, pt6 = 1) or as Ref. Right, Mut. nuclei percentages per cell type (top) and across cell types (bottom). **b**, Representative images of co-immunofluorescence staining on formalin-fixed paraffin-embedded sections (*n* = 1 per patient) showing mTOR-hyperactive (pS6^+^) neurons (NEUN^+^, pt2), astrocytes (GFAP^+^, pt2), oligodendrocytes (OLIG2^+^, pt2) and microglia (IBA1^+^, pt10). Nuclei (in blue) are labeled with DAPI. Scale bars, 20 µm. All patients included in this experiment are detailed in Supplementary Table 2. **c**, Cytomegalic cells representing a minor fraction of mutated cells. Left, representative immunostaining of SMI311^+^ DNs and VIM^+^ BCs on frozen brain tissue from pt5. Nuclei (in blue) are labeled with DAPI for total cell counting. Scale bar, 25 µm. Right, mutated cell percentage (inferred by the detected VAF) and proportion of DNs or BCs identified in each patient (*n* = 1 section/patient/staining was analyzed). **d**, Schematic of the distribution of mutated cells across cell types and the fraction of mutated cytomegalic cells in pt10. Astro, astrocytes; Endo, endothelial cells; Hemi, hemispherical; Oligo, oligodendrocytes; Micro, microglia.

**Extended Data Fig. 4:**
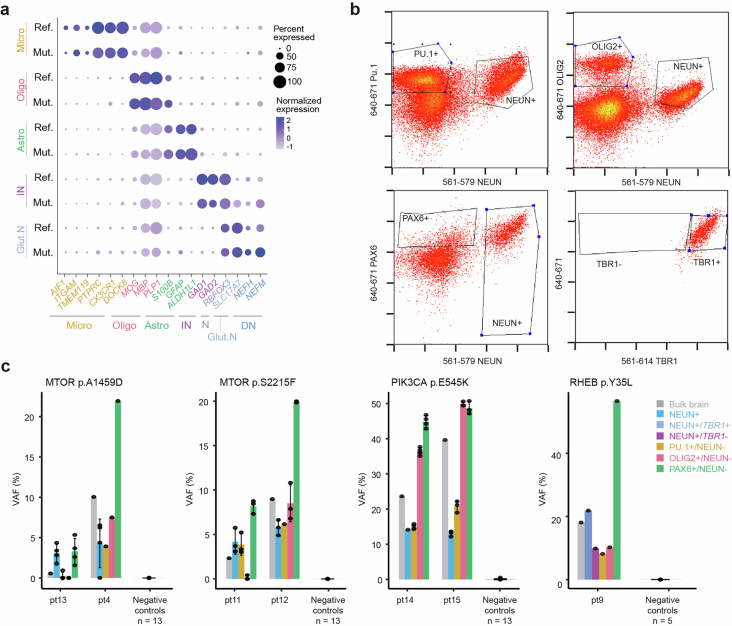
Distribution of somatic mutations across cell types in FCDII. **a**, Expression analysis of cell-type-specific markers in mutation detected (Mut.) and reference detected (Ref.) nuclei. Dot size represents the proportion of nuclei expressing each marker; color intensity indicates average normalized expression level. Abbreviations: N, neurons; Glut.N, glutamatergic neurons; IN, interneurons; Astro, astrocytes; Oligo, oligodendrocytes; Micro, microglia. **b**, Fluorescence-activated nuclei sorting (FANS) gating strategy for cell population enrichment. Representative gating from pt9 is shown. Sequential gating begins with initial selection based on DAPI nuclear staining, followed by separation of neuronal (NEUN+) and non-neuronal populations. Cell-type-specific enrichment was then achieved using TBR1 for glutamatergic neurons, PAX6+/NEUN- for astrocytes, OLIG2+/NEUN- for oligodendrocytes, and PU.1 + /NEUN- for microglia. Note: TBR1 subpopulation analysis was only performed for pt9 due to tissue constraints. **c**, Quantification of somatic mutations across FANS-enriched cell populations was performed using two complementary approaches: ddPCR detection for *MTOR* and *PIK3CA* variants, and deep targeted amplicon sequencing (TAS) for the *RHEB* variant in pt9. Mutation-positive bulk brain DNA and mutation-negative blood DNA served as controls. ddPCR detection limits (LOD) were >3 FAM+ mutated droplets for *MTOR*, >6 FAM+ mutated droplets for *PIK3CA* in ddPCR analysis. Results are presented as mean ± SD where technical replication was feasible. Due to limited tissue availability, biological replicates could not be performed, and technical replicates were not possible for specific cell populations in patients pt4 (PAX6+/NEUN- and OLIG2+/NEUN-), pt9 (none), pt12 (PU.1+/NEUN-), and pt14 (PU.1+/NEUN-).

We next investigated which cell types carry FCDII-causing somatic mutations and how this affects gene expression. To determine genotypes at single-cell resolution, we initially combined 3′-snRNA-seq data and PacBio-targeted long-read sequencing of barcoded snRNA-seq transcripts. Only *RHEB* variants showed sufficient coverage among mutation sites, as previously reported^23^. We classified nuclei as mutation detected (Mut.) or reference detected (Ref.). We genotyped 808 nuclei, predominantly from patients with *RHEB* mutations: patient 10 (pt10; 78.3%) and pt9 (6.4%), with smaller contributions from pt8 (11.4%), pt7 (1.7%), pt6 (1.5%), pt2 (0.4%) and pt4 (0.2%) (Supplementary Table 5). Control snRNA-seq datasets showed only wild-type reads at mutation sites, confirming the absence of false-positive reads (Supplementary Table 6). The proportion of Mut. nuclei (117 Mut. nuclei, ~15%) was within the range of the VAF in bulk brain tissue (9% in pt10), supporting reliable mutation detection. These Mut. nuclei were distributed across various cell types, with enrichment in glutamatergic neurons (25% GluL2/3 (glutamatergic neurons of layers 2 and 3) and 22% GluNs) and astrocytes (22%) (Fig. 2a). A fraction of Mut. nuclei was also assigned to GABA-ergic interneurons (16% CGE derived and 6% MGE derived), oligodendrocytes (13%), microglia (11%) and OPCs (10%). Cell-type identity of Mut. nuclei was confirmed using canonical cell-type-specific markers, excluding putative annotation errors (Extended Data Fig. 4a). Sequencing coverage was uniform across cell types (Supplementary Table 7) and the distribution of Mut. nuclei was significantly different from random distributions (*χ*^2^ = 91.625, *P* < 0.0001; Methods). The mTOR pathway activation was validated by pS6 immunoreactivity in neurons and a subset of astrocytes, oligodendrocytes and microglial cells (Fig. 2b).

To overcome the limitations of genotyping from snRNA-seq data, we next conducted fluorescence-activated nuclear sorting (FANS) with droplet digital PCR (ddPCR) and targeted deep amplicon DNA sequencing to identify the somatic mutations in enriched cell populations. This step served to provide orthogonal validation for the distribution of somatic variants across distinct nuclei-sorted populations and to extend our findings to a larger cohort of seven patients with FCDII (pt4 and pt9 from the initial cohort and five additional cases with mutations in *MTOR* and *PIK3CA*; details of individuals included in each experiment are in Supplementary Tables 1 and 2). FANS analysis confirmed consistent mutation presence in neuron-enriched (NEUN^+^) and astrocyte-enriched (PAX6^+^/NEUN^−^) populations in all patients, as well as in oligodendrocyte- or OPC-enriched samples (OLIG2^+^/NEUN^−^) in five of seven patients and in microglia-enriched samples (PU.1^+^/NEUN^−^) in six of seven patients. The cell-type distribution pattern varied across patients, for example, *PIK3CA*-mutated hemimegalencephaly cases with high mosaicism showed mutation enrichment in glial cells (astrocytes and oligodendrocytes), whereas *MTOR*-mutated FCDII cases with low mosaicism showed predominant neuronal and astrocytic distribution (Extended Data Fig. 4b,c and Supplementary Table 8). Overall, snRNA-seq and FANS approaches demonstrated broad distribution of somatic mutations across neural cell lineages, extending our initial findings from *RHEB* to *MTOR* and *PIK3CA* mutations.

The crucial role of the mTOR pathway in regulating cell growth raises the question of the extent to which mutated cells exhibit a cytomegalic phenotype in FCDII. We therefore examined what proportion of mutated cells displayed cytomegalic features. For each patient, we compared the fraction of DNs or BCs with that of mutated cells (estimated from the VAF of the somatic variant in bulk tissue). DNs and BCs were identified using SMI311 and VIM markers, respectively, and by their large soma (≥20 µm in diameter; Fig. 2c). Only 1–10% of mutated cells were DNs or BCs (Fig. 2c,d), representing 0.3–0.8% of all cells within the resected FCDII tissues (Supplementary Table 9). This low abundance likely explains their absence as distinct cell clusters in the UMAP (Fig. 1d) despite their striking abnormal morphological appearance. Hence, although mTOR-activating mutations can occur in distinct cell types, only a small fraction develop the cytomegalic phenotype characteristic of DNs or BCs, indicating that mTOR hyperactivation can occur in the absence of increased cell size.

### Cell-type-specific transcriptional effects of FCDII mutations

**Fig. 3:**
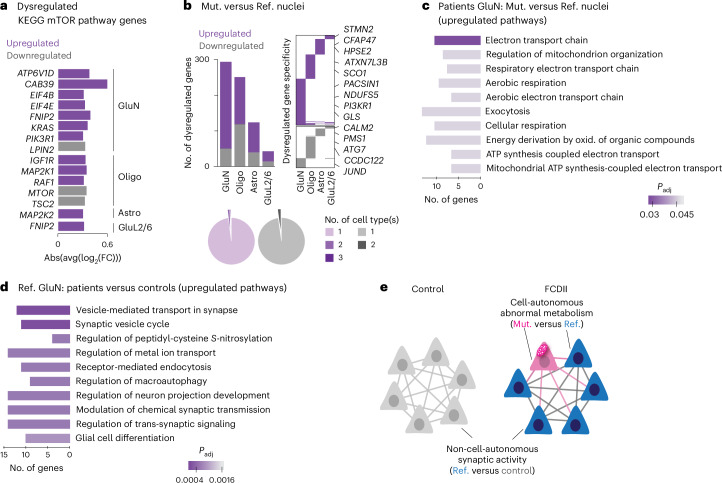
Cell-type-specific transcriptional dysregulation in FCDII mutated cells. **a**,**b**, Cell-type-specific differential gene expression analysis between Mut. and Ref. nuclei from pt9 and pt10 (providing the highest numbers of genotyped nuclei). **a**, Absolute average log_2_(FC) values for dysregulated genes belonging to the mTOR pathway (KEGG database) across cell types. **b**, Top left, dysregulated gene counts per cell type (expressed in 25% of cells with absolute log_2_(FC) > 0.3). Top right, specific and shared dysregulated genes across cell types. Bottom, proportion of dysregulated genes specific to one or more cell types. **c**,**d**, Top GO terms (based on *P*_adj_ values) in Mut. versus Ref. GluNs from patients (**c**) and in Ref. GluNs from patients (pt9 and pt10) versus age-matched GluNs from control samples (ct1 and ct2) (**d**). **e**, Schematic showing cell-autonomous metabolic alterations and non-cell-autonomous synaptic activity changes in FCDII. oxid., oxidation.

**Extended Data Fig. 5:**
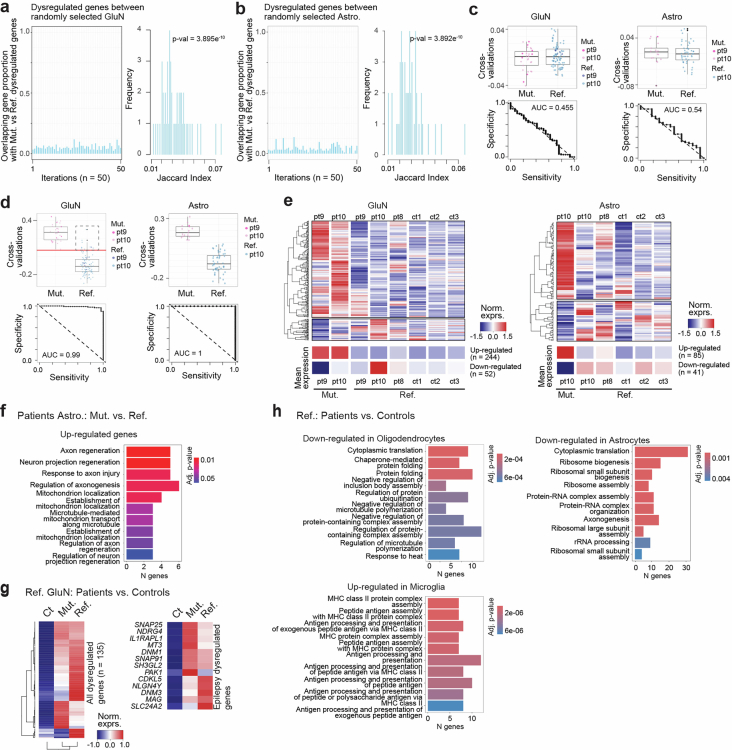
Cell-type-specific transcriptional changes in mutation-carrying cells across FCDII tissues. **a-e**, Validation of transcriptional changes (that is genes with absolute log_2_(FC) > 0.3) between mutation detected (Mut.) and reference detected (Ref.) glutamatergic neurons (GluN) and astrocytes (Astro). Box plots depict the median and interquartile range, with whiskers indicating minimum and maximum values. **a-b**, Differential expression analysis using 50 random subset comparisons of n = 29 GluN (**a**) or n = 17 astrocytes (**b**) amongst Mut. and Ref. nuclei of pt9 and pt10 compared to the rest of GluN and astrocytes, respectively. Left, proportion of shared dysregulated genes in ‘random’ vs ‘observed’ Mut. vs Ref. comparisons. Right, Jaccard similarity index. Wilcoxon signed rank test with continuity correction confirms significant differences between ‘random’ and ‘observed’ dysregulated genes in both GluN and astrocytes. **c**, Linear regression model using ‘random’ dysregulated genes from iteration n.1 of GluN and Astro cannot predict Mut. vs Ref. nuclei. Box plots depict the median and interquartile range, with whiskers indicating minimum and maximum values. **d**, Linear regression model using observed dysregulated genes successfully discriminates Mut. from Ref. nuclei for both GluN and Astro, with potential false negatives identified in 14% (1/7) of pt9 and 9% (8/85) of pt10 Ref. GluN nuclei (indicated by black dotted box above red threshold line). Box plots depict the median and interquartile range, with whiskers indicating minimum and maximum values. **e**, Expression patterns of GluN and Astro ‘observed’ dysregulated genes across patients (pt9-10) and controls (ct1-3). Analysis restricted to samples with >10 Mut./Ref. nuclei. Statistical analysis of average gene expression in Mut. vs. Ref. GluN by individual using Kruskal-Wallis test shows significant mutation effects (genes upregulated in Mut.: H statistics = 340.50, FDR-adjusted p-value: 9.92×10 − 76; genes downregulated in Mut.: H statistics = 30.59, FDR-adjusted p-value: 3.19×10 − 8). No statistical test was performed for astrocytes since only one patient with > 10 Mut. nuclei was available. **f**, Top 10 GO terms for Mut. vs. Ref. dysregulated genes in astrocytes. **g**, Mean expression of GluN dysregulated genes between Ref. nuclei from patients vs. controls in Mut., Ref. and control GluN from pt9-10 and ct1-2. Left, all dysregulated genes. Right, epilepsy-related dysregulated genes. **h**, Top 10 GO terms for patient Ref. vs. control. nuclei dysregulated genes. Only significant GO terms (adjusted p-value < 0.05) are shown.

To identify the cell-autonomous pathways affected by mTOR-activating mutations on gene expression, we compared patients’ Mut. nuclei with nuclei in which only the reference alleles were detected (Ref.), limiting the analysis to cell lineages with at least ten mutated cells: glutamatergic neurons (GluNs and GluL2/6 clusters), astrocytes and oligodendrocytes. Fourteen transcripts were annotated as part of the mTOR pathway in the Kyoto Encyclopedia of Genes and Genomes (KEGG) database (Fig. 3a). Glutamatergic neurons displayed the highest number of dysregulated genes, although no single gene was statistically differentially expressed between Mut. and Ref. cells, probably as a result of the limited number of nuclei analyzed (Fig. 3b, Supplementary Table 10 and Extended Data Fig. 5a–d). Supporting genuine biological differences, however, GO term analysis revealed meaningful and statistically significant differences across cell types. Within Mut. and Ref. nuclei, enriched GO terms were related to mitochondrial function and organization, metabolism, respiration and exocytosis. Similarly, mitochondrial metabolism and transport-related GO terms were enriched in mutated astrocytes, whereas oligodendrocytes did not show statistically significant GO term enrichment (Fig. 3c, Supplementary Table 10 and Extended Data Fig. 5f). These findings support cell-type-specific alterations in metabolic pathways in mutated neurons and astrocytes.

We further observed that the expression level of these dysregulated genes was similar in Ref. nuclei from patients and controls (Extended Data Fig. 5e), suggesting that the transcriptional changes are specific to mutated nuclei and consistent across individuals despite the limited number of nuclei analyzed (Supplementary Table 10).

Non-cell-autonomous mechanisms causing hyperexcitability of nonmutated neurons located close to mutated ones have also been described in an FCDII mouse model^24^. To investigate the potential effects of mutated cells on their neighbors, we compared Ref. GluNs from patients with GluNs from controls. We identified upregulation of genes involved in synaptic transmission and regulation of neuron projection development (Fig. 3d and Supplementary Table 11); ~90% of these genes were also upregulated in Mut. GluNs compared with control nuclei (Extended Data Fig. 5g). This finding indicates widespread synaptic pathway changes affecting both mutated and nonmutated neurons. Similar non-cell-autonomous effects were observed in glial cells (Extended Data Fig. 5h). Together, these observations indicate two processes in FCDII: (1) cell-autonomous mechanisms in mutated cells affecting mitochondria function and (2) broader circuit-level changes affecting both mutated and nonmutated neurons (Fig. 3e).

### DNs and BCs have distinct molecular identities

DNs and BCs can be identified by histopathological markers such as SMI311 and VIM^15^, but their comprehensive molecular identity remains unexplored. To address this, we applied multiple complementary approaches to determine the transcriptional identity of DNs and BCs: laser capture microdissection with transcriptome-wide profiling (LCM–seq), Visium spatial transcriptomics for whole-transcriptome analysis and MERSCOPE for high-resolution targeted gene profiling.

**Fig. 4:**
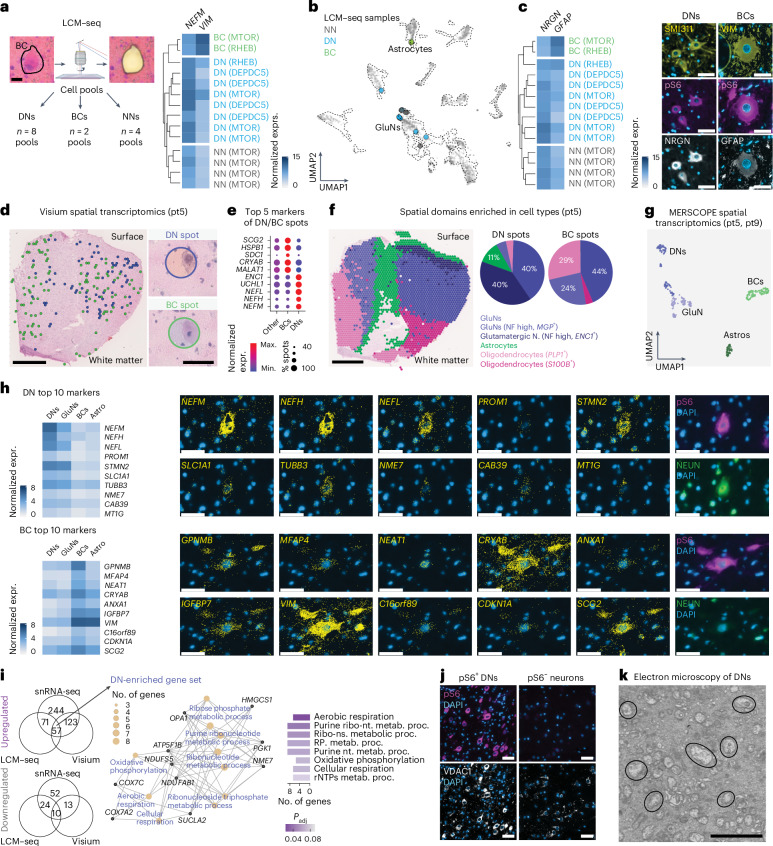
DNs and BCs belong to glutamatergic and astroglial lineages and display metabolic dysregulations. **a**, Left, LCM–seq workflow for capturing pools of DNs, BCs and NNs from eight patients (pt1–5 and pt7–9). Right, heatmap of *NEFM* and *VIM* normalized expression with unsupervised hierarchical clustering. **b**, Label transfer of LCM–seq samples on to the snRNA-seq UMAP space showing NNs or DNs matching with GluNs and BCs with astrocytes. **c**, Left, *NRGN* and *GFAP* normalized expression heatmap with unsupervised hierarchical clustering. Right, co-immunofluorescence showing NRGN in pS6^+^/SMI311^+^ DNs and GFAP in pS6^+^/VIM^+^ BCs (pt5) (*n* = 1 section/patient/staining analyzed). GFAP-pS6 and VIM-pS6 double stainings were performed on two consecutive sections and the same BC was recognized in both sections. Nuclei (in blue) are labeled with DAPI. Scale bars, 50 µm. **d**, Visium spatial transcriptomics showing intermingled spots containing DNs and BCs across the tissue (pt5). Magnified images show representative DN- and BC-containing spots after hematoxylin and eosin staining (*n* = 1 section per patient analyzed). Scale bars, 1.5 mm; insets = 55 µm. **e**, Top markers of DN- and BC-containing spots (pt5). Known histological markers for DNs (*NEFM*) and BCs (*CRYAB*) are enriched in spots with DNs and BCs. **f**, Spatial semi-supervised clustering of Visium spots showing clusters enriched in GluNs, astrocytes and oligodendrocytes (pt5) with top marker genes in parentheses. **g**, Distinct clusters for DNs, BCs, astrocytes (Astros) and GluNs from single cells (pt5 and pt9) of the MERSCOPE UMAP space. **h**, Heatmap of the top ten DN or BC markers with representative MERSCOPE images (pt5). DNs are identified as pS6^+^/NEUN^+^ and BCs as pS6^+^/NEUN^−^ (*n* = 1 section per patient analyzed). Scale bars, 50 µm. **i**, Left, number of shared dysregulated genes across Mut. versus Ref. GluNs (snRNA-seq), DNs versus NNs (LCM–seq) and DN-containing spots (Visium). Right, top GO terms of DN upregulated genes. Ribo-nt., ribonucleotides; metab., metabolic; proc., process; Ribo-ns., ribonucleosides; RP., ribosomal proteins; rNTP, ribonucleoside triphosphates. **j**, Representative images of strong VDAC1 immunostaining in pS6^+^ DNs (pt2) (*n* = 1 section/patient/staining analyzed). Scale bars, 50 µm. **k**, Electron microscopy of DNs (pt5) showing an accumulation of vesicular, swollen, damaged mitochondria (black circles) (*n* = 1 section per patient analyzed). Scale bar, 2.5 µm. Detailed sample information for each experiment and analysis is provided in Supplementary Table 2. expr., expression; max., maximum; min., minimum.

**Extended Data Fig. 6:**
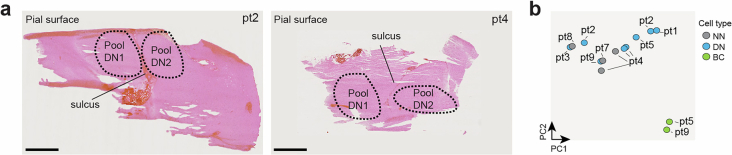
Laser capture microdissection sequencing (LCM-seq) analysis of FCDII tissues. **a**, Hematoxylin and eosin (HE) staining of 20μm-thick frozen sections (n = 1 for each patient) adjacent to those used for laser capture microdissection, showing two distinct gyri where dysmorphic neuron (DN) pools were collected from patients pt2 and pt4. Scale bar = 2.5 mm. **b**, 2D Principal component analysis (PCA) of LCM-seq samples based on the top 2,000 variable genes comparing normal neurons (NN), dysmorphic neurons (DN) and balloon cells (BC), showing distinct clustering of BC.

First, we performed full-length SmartSeq mRNA sequencing of laser-captured pools of 160 DNs (*n* = 8), BCs (*n* = 2) and normal-appearing neurons (NNs, *n* = 4), selected based on morphology and soma size (Fig. 4a, Extended Data Fig. 6a and Supplementary Table 12). The *RHEB* somatic mutation site was present in ~50% of reads from both DNs and BCs, as expected. Principal component and hierarchical cluster analyses on the top 2,000 variable genes indicated that BCs (expressing high levels of *VIM*) clustered separately from NNs and DNs (expressing high levels of *NEFM*) (Fig. 4a and Extended Data Fig. 6b). Using label transfer, we integrated these cell pools into the snRNA-seq UMAP space to identify the nearest cell-type identities. DNs and NNs mapped to GluNs, whereas BCs clustered with astrocytes (Fig. 4b). Accordingly, immunofluorescence staining revealed that DNs (identified as pS6^+^/SMI311^+^) expressed the GluN-enriched protein neurogranin (NRGN) and BCs (identified as pS6^+^/VIM^+^) expressed the astrocytic marker glial fibrillary acidic protein (GFAP) (Fig. 4c).

**Extended Data Fig. 7:**
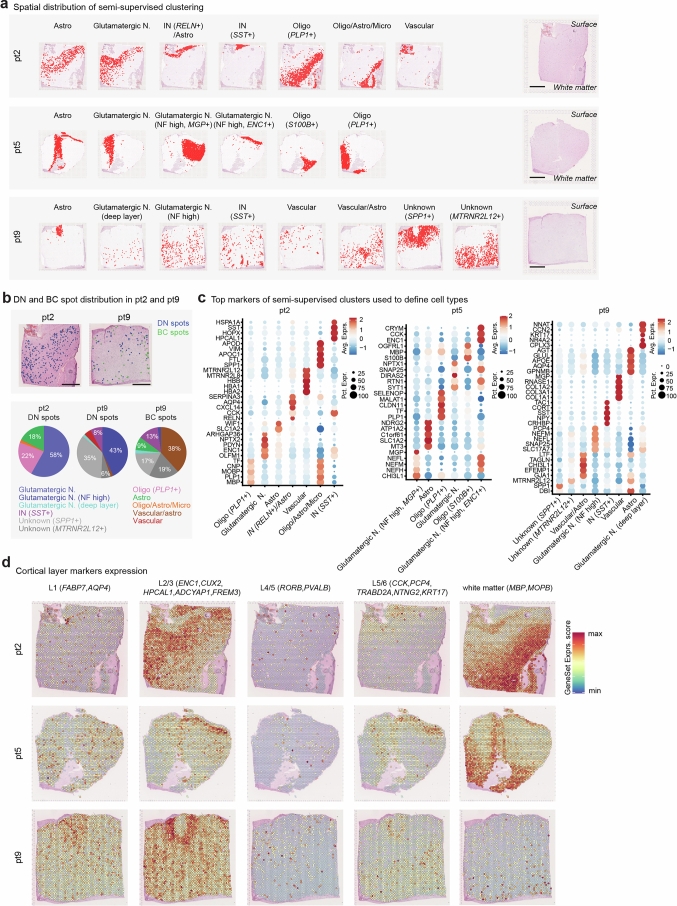
Visium spatial transcriptomics of FCDII postoperative brain tissues. **a**, Semi-supervised clustering of Visium data from patients pt2, pt5 and pt9. Clusters (highlighted in red) are annotated based on predominant marker genes. To differentiate clusters enriched for the same cell type, the top expressed marker was added to the cluster annotation. Clusters lacking clear cell-type enrichment in pt9 are labeled ‘Unknown’. Abbreviations: N., neurons; IN, interneurons; NF high, cluster with high levels of neurofilament genes; Astro, astrocytes; Oligo, oligodendrocytes; Micro, microglia. On the right: corresponding hematoxylin and eosin (HE) -stained section. Scale bars = 1.5 mm. **b**, Spatial distribution of DN/BC-containing spots in pt2 and pt9 showing distribution across both anatomical space and transcriptionally-defined clusters. To differentiate clusters enriched for the same cell type, the top discriminating marker expressed was added to the cluster annotation. Scale bars = 1.5 mm. **c**, Expression analysis of cluster-defining markers in each Visium sample. Dot size indicates percentage of expressing spots; color intensity shows average normalized expression. Abbreviations: Pct. Expr., percentage of spots expressing the genes; Avg. Exprs., average normalized gene expression per group. **d**, Spatial distribution of cortical layer marker expression scores calculated using previously defined gene sets (Maynard et al., 2021)^50^. Higher scores indicate increased expression of the gene set.

To incorporate spatial resolution into our analysis, we performed Visium spatial transcriptomics on frozen cortical sections from three patients (pt2, pt5 and pt9). We analyzed an average of 2,992 spots per sample, with each spot containing between 0 and 8 cells (mean of 4–5 cells) and an average of 2,846 genes. Across all samples, we morphologically identified 475 spots containing DNs (which expressed high levels of the DN marker neurofilament-encoding genes *NEFH*/-*M*/-*L*) and 159 spots containing BCs (which expressed the BC marker *CRYAB*) (Fig. 4d,e). Semi-supervised clustering revealed distinct spatial distributions of spots containing DNs and BCs across tissues (Fig. 4f; Extended Data Fig. 7). DN-containing spots were predominantly located among glutamatergic neurons throughout all cortical layers. In contrast, BC-containing spots were dispersed throughout the tissue. These findings further support the hypothesis that DNs relate to glutamatergic neurons, whereas BC cells distribute throughout the white and gray matter in an astroglial-like pattern.

**Extended Data Fig. 8:**
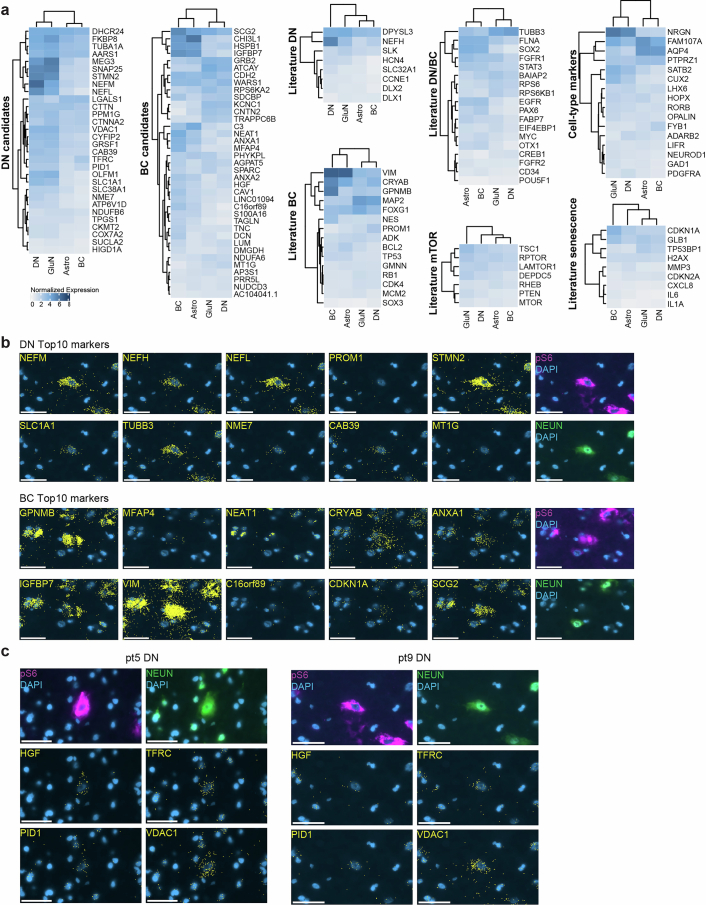
MERSCOPE gene panel analysis of FCDII postoperative brain tissues. **a**, Heatmaps showing normalized (SCTransform) gene expression levels of the 140-gene MERSCOPE panel, grouped by category. Abbreviations: DN, dysmorphic neurons; BC, balloon cells. **b**, Representative MERSCOPE images of top expressed genes (from Fig. 4h) in DN (pS6+/NEUN+) and BC (pS6+/NEUN-) from patient pt9 (n = 1 tissue section examined). Yellow puncta indicate individual transcript molecules. Scale bar = 50μm. **c**, Spatial detection of mitochondrial gene transcripts (yellow puncta) in DN from patients pt5 and pt9 (n = 1 tissue section examined for each patient). Scale bar = 50 μm.

Integration of LCM–seq, Visium and snRNA-seq data revealed distinct gene expression signatures in DNs and BCs. Both cell types expressed mitochondria-associated genes (*COX7A* and *VDAC1* in DNs, *AGPAT5* in BCs). DNs specifically expressed genes related to neurotransmitter transport (*SNAP25*, *SLC38A1* and *SLC1A1*) and apoptosis (*OLFM1*, *LGALS1* and *TFRC*), whereas BCs expressed genes involved in vesicle organization (*TRAPPC6B*, *CAV1*, *ANXA1* and *ANXA2*) and cell migration (*SDCBP*, *HGF* and *TNC*). To validate these findings at higher resolution, we designed a 140-gene MERSCOPE spatial transcriptomics panel, including canonical cell-type markers, known DN or BC markers and some of our newly identified DN or BC candidate markers (Supplementary Table 13). We analyzed frozen cortical sections from two patients with *MTOR* (pt5) and *RHEB* (pt9) somatic mutations and identified DNs and BCs based on pS6 and NEUN protein immunofluorescence. As a result of the transcriptional proximity of DNs and BCs to GluNs and astrocytes, respectively, we compared gene expression across DNs (cytomegalic pS6^+^/NEUN^+^), BCs (cytomegalic pS6^+^/NEUN^−^), normal-sized GluNs (pS6^−^/NEUN^+^-expressing *NRGN*) and astrocytes (pS6^−^/NEUN^−^-expressing *AQP4*). Using this approach, we observed that DN and BC clusters were distinct from GluNs and astrocytes in the UMAP space, indicating that our customized gene panel is suitable for differentiating cytomegalic DNs or BCs from other cells in FCDII tissues (Fig. 4g). We validated 22 of 41 known histological DN or BC markers at the transcriptional level (*f*or example, for DNs: increased expression of *NEFH* and *SLC32A1* and decreased expression of *SLK*; for BCs: increased expression of *VIM*, *CRYAB* and *GPNMB*) (Extended Data Fig. 8a). Among the top ten genes driving the DN and BC clustering, we found six known histological markers (*NEFM*/-*H*/-*L*, *GPNMB*, *VIM* and *CRYAB*) and identified new candidates, such as *SLC1A1* (glutamate transporter) and *STMN2* (microtubule stability regulator) for DNs or *MFAP4* (extracellular matrix protein enriched in progenitor cells) and *IGFBP7* (insulin-like growth factor-binding protein 7 associated with cellular senescence) for BCs (Fig. 4h and Extended Data Fig. 8b). MERSCOPE data confirmed the expression of mitochondrial genes in DNs (for example, *HGF*, *PID1*, *TFRC* and *VDAC1*) (Fig. 3c and Extended Data Fig. 8c). Furthermore, we identified 57 upregulated genes in DNs through the intersection of LCM–seq, Visium and snRNA-seq (Mut. versus Ref. GluNs) datasets (Supplementary Table 14). Pathway analysis of these genes revealed enrichment in metabolism, mitochondrial function, cell respiration and ribonucleotide metabolism across patients with mutations in *MTOR*, *RHEB* and *DEPDC5* (Fig. 4i).

**Extended Data Fig. 9:**
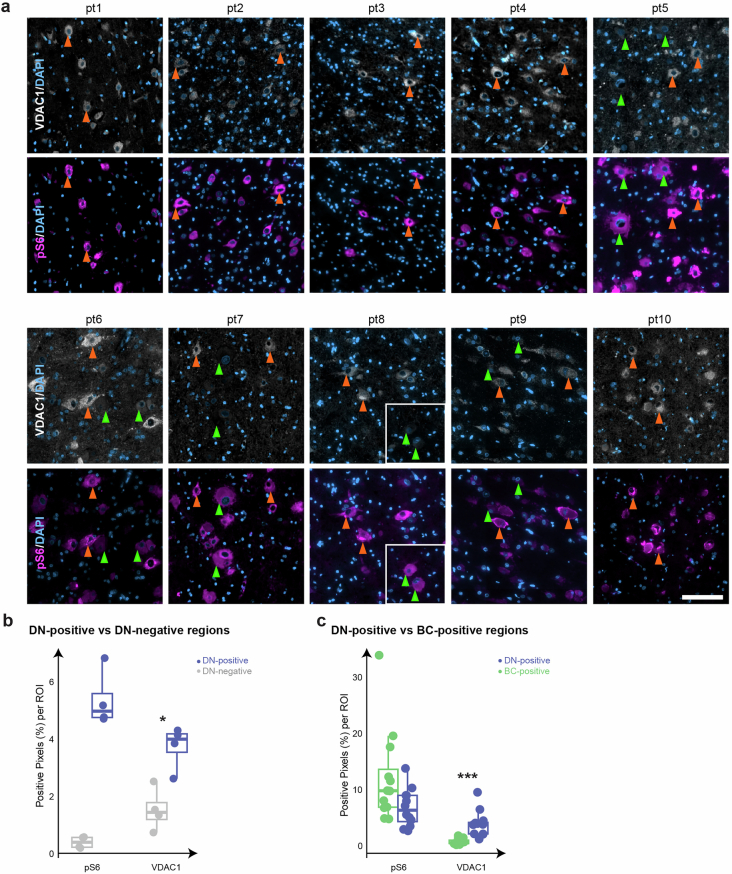
VDAC1 and pS6 co-immunofluorescence in dysmorphic neurons. **a**, Co-immunofluorescence analysis of VDAC1 (white) and pS6 (pink) across FCDII specimens (patients pt1-10, n = 1 section/patient). Strong VDAC1 signal is observed in dysmorphic neurons (DN, enlarged pS6+ cells, orange arrowheads), while balloon cells (BC, pS6+ cells with glassy cytoplasm, green arrowheads) display weak VDAC1 signal. Inset in pt8 shows representative BC from distinct region. Scale bar = 100 μm. **b**, Quantitative comparison of VDAC1 expression between DN-positive and DN-negative regions in pt2. Analysis was performed on 0.25 mm² regions of interest (ROIs, n = 4 per condition), showing percentage of VDAC1+ and pS6+ pixels. Two-sided Wilcoxon rank sum exact p-value = 0.02857*. Box plots show median, interquartile range, and min/max values. **c**, Quantification of VDAC1 expression between DN-positive and BC-positive regions in pt5-8. Analysis was performed on 0.25 mm^2^ ROIs (n = 3 per region per patient; total n = 12 per condition). Data shown as percentage of VDAC1+ pixels and pS6+ pixels per ROI. Two-sided Wilcoxon rank sum test, exact p-value = 5.177e-06***. Box plots depict the median and interquartile range, with whiskers indicating minimum and maximum values.

Given that these mitochondrial transcriptional alterations were initially identified from a limited set of genotyped nuclei, primarily from one *RHEB*-mutated patient, we sought to validate our findings at the protein and structural levels across the entire patient cohort. We observed strong VDAC1 immunoreactivity, a marker of mitochondrial biomass, in pS6^+^ DNs in all patients tested (pt1–10), whereas BCs showed weak immunoreactivity (Fig. 4j and Extended Data Fig. 9), confirming transcriptional upregulation in DN.

**Extended Data Fig. 10:**
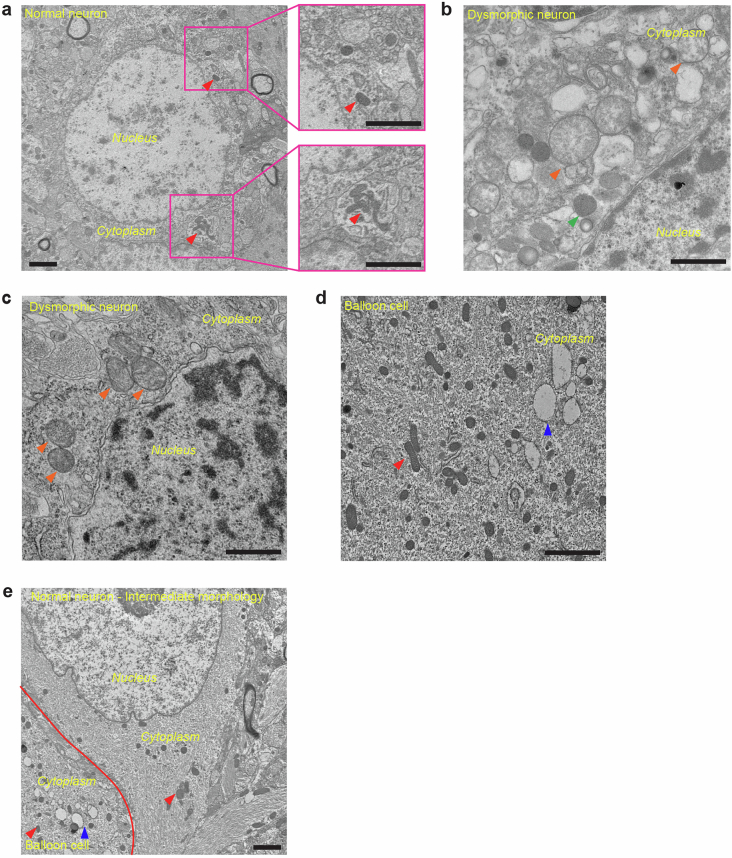
Ultrastructural analysis of FCDII human tissue by electron microscopy. **a-e**, Pyramidal neurons with normal morphology and abnormal cytomegalic dysmorphic neurons or balloon cells were identified in electron microscopy samples from patients pt4, pt5 and pt7 (n = 1 section/patient). **a**, Normal pyramidal neuron from pt5 showing mitochondria with typical morphology (red arrowheads). Scale bar = 2μm. **b**, Dysmorphic neuron (DN) from pt4 displaying structurally damaged swollen mitochondria (orange arrowheads) and lysosomes with heterogenous appearance (green arrowheads). Scale bar = 1μm. **c**, DN from pt7 displaying structurally damaged swollen mitochondria (orange arrowheads). Scale bar = 1μm. **d**, Balloon cell (BC) from pt5 characterized by accumulation of normal morphology mitochondria (red arrowheads), vacuoles (blue arrowheads) and cytoplasm filled with intermediate filaments. Scale bar = 2μm. **e**, Normal mitochondria (red arrowheads) and cytoplasmic accumulation of neurofilaments in a non-cytomegalic neuron with abnormal intermediate phenotype from pt5 adjacent to a BC with normal mitochondria (red arrowheads) and vacuoles (blue arrowheads) in the cytoplasm. Scale bar = 2μm.

Ultrastructural examination of DNs from three FCDII tissues by electron microscopy revealed cytoplasmic accumulation of intermediate filaments (probably neurofilament) and structurally damaged mitochondria with a vesicular, swollen morphology (and only occasional morphologically normal mitochondria). In contrast, normal pyramidal neurons and BCs contained mostly mitochondria of normal morphology (Fig. 4k and Extended Data Fig. 10).

Overall, these findings using multiple analytical approaches suggest an accumulation of damaged mitochondria resulting from the presence of mTOR-hyperactivating variants.

## Discussion

In the present study, using comprehensive identity profiling at the genomic, transcriptomic and morphological levels, we uncovered the identity of pathogenic cytomegalic cells in FCDII and the autonomous and non-cell-autonomous effects of somatic mutations on these cells and the neural circuit. Our findings reveal that DNs are molecularly closely related to glutamatergic neurons, whereas BCs more closely resemble astrocytes. Hence, rather than representing different states of a single-cell type, DNs and BCs are distinct cell types that probably arose from a single mutational event before the divergence of neuronal and astroglial lineages. DNs and BCs, which are very rare in brain tissues, did not form distinct FCDII-specific clusters in our snRNA-seq analysis, consistent with a preprint study^25^, suggesting that transcriptional dysregulation of these cells does not overtly affect cell-type identity.

DNs exhibit characteristics of molecularly dysfunctional glutamatergic neurons: they express high levels of neurofilaments, lack specific cortical layer markers, display mitochondrial abnormalities and show altered synaptic gene expression that may drive their intrinsic excitability^8^. Our analysis reveals that DNs and BCs probably contribute to epileptogenesis through distinct mechanisms: DNs display molecular hallmarks of hyperexcitability (for example, increase of the glutamate transporter *SLC1A1*) and may thus affect nonmutated circuit partners through synaptic transmission changes, whereas BCs display high expression of genes coding for secreted proteins (for example, *MFAP4* and *IGFBP7*) that may affect neighboring cells in a paracrine manner. Expression of *IGFBP7*, a component of the senescence-associated secretory phenotype, further supports a critical role for cellular senescence in FCDII, as recently reported^26^. Notably, although both DNs and BCs consistently show increased mTOR pathway activation (indicated by pS6 protein levels), few transcripts of the pathway were dysregulated in these cells, indicating that mTOR hyperactivation is not primarily driven by transcriptional changes.

Remarkably, our findings reveal that mTOR-activating mutations lead to cytomegaly in only a minority of cells in FCDII tissues. Specifically, DNs and BCs represent <10% of mutated cells, suggesting that they are the visible ‘tip of the iceberg’ of a much larger population of mutated cells in FCDII tissues. This observation challenges previous assumptions that DNs and BCs are the primary contributors to the VAF of somatic mutations in bulk brain tissues. Other mutated cells, although not meeting the criteria for a pathological hallmark cell, may exhibit various alterations such as misorientation and mislocalization (potentially contributing to FCDII dyslamination) or abnormal electrophysiological properties without overt morphological changes. Our findings contrast with in utero electroporation-based mouse models of FCDII, in which electroporated cells consistently display increased cell size^27^. This difference may reflect variations in mTOR hyperactivation levels, as mutant proteins are overexpressed in mouse models, or differences in how human and rodent neural progenitors respond to mTOR pathway activation. The molecular mechanisms underlying this variability in cellular responses to mTOR activation remain unknown but may depend on factors such as mutation timing, cell-type-specific elements or non-cell-autonomous influences, as evidenced by a recent study showing that wild-type cells can suppress aberrant growth induced by oncogenic *Ras* mutations^28^.

Our comprehensive genotyping approach proceeded in two steps. First, using transcriptome-based methods (snRNA-seq and cDNA long-read sequencing), we revealed mutation distribution in distinct cell types in two *RHEB*-mutated specimens with large dysplasia and high VAF. We then validated and extended these findings through FANS and targeted sequencing of genomic DNA in five additional FCDII cases with *MTOR* and *PIK3CA* mutations. Mutations were consistently detected in neurons and astrocytes, with variable presence and proportions in oligodendrocytes and microglia. The presence of mutation in cortical GABA-ergic neurons, canonically originating from the ganglionic eminences, provides support for recent studies reporting a dorsal origin for some interneurons^29–32^. While our analysis also detected mutations in a few microglia, which derive from the mesoderm, this finding—if confirmed—would suggest very early mutational events that presumably occurred before the divergence of these cell lineages (that is, possibly during gastrulation, at gestational weeks 2–3, when the neuroectoderm forms)^33^. Our findings are consistent with a recent snRNA-seq study that reported a high-VAF somatic *RHEB* mutation distributed across various brain cell types in a patient with FCDII, suggesting a pre-gastrulation mutational event^23^. Nevertheless, it is possible that low VAF (~1%) variants in small FCDII lesions occur at later developmental stages and therefore present with a more restricted cell-type distribution. The variable distribution of mutated cells across cell lineages and across patients may reflect heterogeneity in mutational timing, extent of the cortical malformation or selective pressures during development.

Although early embryonic mutational events, in principle, affect multiple organs^34^, FCDII somatic mutations are typically found only in the brain, not in the blood or saliva. Negative selection of mutated cells outside of the central nervous system might explain this finding, as recently reported for cortical malformations associated with somatic gain of chromosome 1q, suggesting a tissue-specific selection process for somatic mutations during development^5^.

Transcriptomic analyses revealed two distinct patterns of gene dysregulation in FCDII tissues. Nonmutated cells showed non-cell-autonomous alterations in pathways related to neurodevelopment, neurotransmission and synapse in glutamatergic neurons. Instead, mutated glutamatergic neurons exhibited cell-autonomous changes in metabolism and mitochondrial function. These mitochondrial alterations were validated across patients with different mTOR pathway mutations using orthogonal approaches, including protein and ultrastructural analyses. These findings parallel observations in tuberous sclerosis complex (TSC), another mTOR-related disorder characterized by DNs and BCs. Specifically, neurons lacking *TSC1*/-*2* (components of an mTOR pathway inhibitory complex) also show accumulation of damaged mitochondria. Recent evidence also indicates increased oxidative stress and neuroinflammation in both patients with FCDII and TSC, suggesting shared mitochondrial dysfunction^35^. Therefore, the accumulation of damaged mitochondria in DNs could result from excessive mTOR activity on mitochondrial biogenesis or fission^36^ and oxidative phosphorylation, leading to free radical accumulation^37^.

To conclude, our study provides key insights into the cellular and molecular complexity of FCDII, implicating altered mitochondrial function in DNs. Hyperactivation of mTOR in FCDII tissues seems to accelerate processes related to cellular aging, such as mitochondrial energy production and cellular senescence, and block the autophagic clearance of damaged mitochondria^26,38^. Senescence- and mitochondria-targeted therapeutics may therefore represent a promising avenue in patients with FCDII, potentially offering an alternative to invasive resective neurosurgery in the future^26^.

## Methods

### Brain samples collection

Written informed consent was obtained from parents or legal guardians of all patients and age-appropriate assent was obtained from patients, when possible, in accordance with an approved Île-de-France II Committee of Protection of Persons protocol (no. ID-RCB/EUDRACT-2015-A00671-48), registered on ClinicalTrials.gov (NCT02890641). The consent covered tissue collection, storage and use for research purposes. The tissue collection and usage from controls adhered to the principles outlined in the Declaration of Helsinki and the Amsterdam UMC Research Code provided by the Medical Ethics Committee (authorization no. W21_295 # 21.326).

Surgical samples were obtained from 15 children (aged from 3 months to 16 years) who underwent epilepsy surgery at the Rothschild Foundation Hospital (Paris, France) between 2016 and 2021. Ten patients underwent resection of the epileptogenic zone (‘focal’ patients) and five underwent a hemispherotomy (functional disconnection of the two hemispheres with resection of frontal cortical tissue; ‘hemispherical’ patients). Both fresh (unfixed) snap-frozen and formalin-fixed paraffin-embedded (FFPE) tissues were obtained for each patient. Nonessential brain tissues, not required for neuropathological diagnosis, were designated for research use. Brain samples were reviewed and classified by board-certified neuropathologists according to the International League Against Epilepsy (ILAE) classification of FCD^7,39^. Cryosections (20 μm thick) from snap-frozen blocks were analyzed by hematoxylin and eosin (H&E) staining. Genomic bulk DNA was extracted from adjacent frozen brain sections and blood samples, according to standard procedures, and bulk RNA was extracted using the Maxwell RSC Tissue RNA kit (Promega) from tissues of patients pt1–10. RNA integrity numbers assessed on a Tapestation (Agilent Technologies) were between 6 and 8.3. Control material was obtained from three, age-matched, postmortem, frontal lobe tissues of individuals without known neurological diseases (ct1–3). The mean age at death was 4.1 years (range 2 months to 10 years), with a mean postmortem interval of 7 h before brain tissue collection (range 6.5–7.5 h). Brain tissue was frozen and stored at −80 °C.

### Genetic investigations

The search for pathogenic mutations was performed by hybrid capture sequencing of bulk brain tissues and blood DNA samples; genetic data were previously reported for patients pt1, pt3–10 and pt12 (refs. ^12,17,40^). For patients pt2, pt11 and pt13–15, a customized panel of ~60 known or candidate focal cortical dysplasia (FCD) genes from Twist Bioscience was used and libraries were sequenced on an Illumina NovaSeq 6000 sequencer. Bioinformatic analysis and validation of the somatic variant were carried out as previously described^12^.

### Immunostaining

To quantify dysmorphic neurons (DNs) and balloon cells (BCs), 20-μm-thick cryosections were obtained from the frozen brain tissue utilized for genetic and RNA investigations of patients pt1–10. Immunohistochemistry was conducted using primary antibodies against VIM (Vimentin-V9, 1:200, Dako, cat. no. M0725) and SMI311 (1:200, BioLegend, cat. no. 837801) and revealed using the POLYVIEW (AP) and HIGHDEF green (anti-mouse) kit (Enzo Life Sciences). Sections were counterstained with DAPI and scanned in both brightfield and fluorescence channels with a Nanozoomer scanner (Hamamatsu) at ×40. Images were exported at ×10 and ×20 magnifications with the NDP.view2 software (Hamamatsu). For immunohistochemistry on 4-μm FFPE sections, a primary antibody against pS6^240/244^ (1:2,000, Cell Signaling, cat. no. 5364) was used, with an avidin–biotin peroxidase complex conjugation system (Vectastain ABC Elite, Vector laboratories) and DAB to detect the biotinylated anti-rabbit secondary antibody (1:250, Vector Laboratory, cat. no. BA-1100). Sections were counterstained with hematoxylin and processed as described above. Co-immunofluorescence was performed using primary antibodies against pS6^240/244^ (1:1,000, Cell Signaling, cat. no. 5364), VIM (1:100, Dako, cat. no. M0725), SMI311 (1:500, BioLegend, cat. no. 837801), GFAP (1:200, Thermo Fisher Scientific, cat. no. MA5-15086), NRGN (1:50, Thermo Fisher Scientific, cat. no. PA5-19209), OLIG2 (1:100, R&D Systems, cat. no. AF2418), IBA1 (1:500, Abcam, cat. no. ab5076), NEUN (1:500, Millipore, cat. no. MAB377) and VDAC1 (1:500, Abcam, cat. no. ab16814). Secondary antibodies included donkey anti-mouse Alexa Fluor-555 (1:1,000, Thermo Fisher Scientific, cat. no. A31570), donkey anti-rabbit Alexa Fluor-647 (1:1,000, Thermo Fisher Scientific, cat. no. A31573) and donkey anti-rabbit Alexa Fluor-488 (1:1,000, Thermo Fisher Scientific, cat. no. A21206). Slides were counterstained with DAPI. Co-immunofluorescence scans were obtained with an Axioscan (Zeiss) and analyzed using Zen 2.3 Blue (Zeiss).

### DN or BC proportion counting in frozen sections

To calculate the proportions of DNs and BCs, we analyzed whole frozen tissue sections stained with SMI311 (for DNs) and VIM (for BCs). Whole-slide scans were imported into QuPath (v.0.3.2) for analysis. The total number of cells per section was determined by automated counting of DAPI^+^ nuclei using QuPath’s cell detection function. DNs and BCs were manually counted across each entire section. The proportion of DNs and BCs was calculated by dividing the total number of DNs or BCs by the total number of DAPI^+^ cells. The number of mutated cells per tissue was estimated based on the variant allele frequency (VAF) from DNA extracted from adjacent tissue sections, ensuring direct correspondence between genetic and histological findings.

### 10× SnRNA-seq

To ensure correlation between histopathology with snRNA-seq data, nuclei were isolated from 150-µm-thick frozen sections adjacent to 20-µm-thick cryosection used for H&E staining. Frozen cortical tissues (40–60 mg) were homogenized using a Wheaton Dounce homogenizer, followed by nucleus isolation through sucrose gradient centrifugation. Subsequently, snRNA-seq was conducted using the droplet-based 10x Chromium platform Next GEM Single Cell 3′ Reagent Kit (v.3.1), targeting 10,000 nuclei per sample. Libraries were sequenced on Illumina HiSeq2500 or Novaseq 6000, targeting 50,000 reads per nucleus. Data processing used Cell Ranger (v.3) with a modified GRCh38 reference genome including intronic regions. Quality controls included: identification of nonempty droplets (DropletUtils R package), presence of <5% of mitochondrial reads, expression between 500 and 12,000 genes and a minimum of 1,000 unique molecule identifiers. To assess the proportion of gray and white matter in the tissues after the experiment, 20-μm-thick cryosections were H&E stained and scanned using a Nanozoomer (Hamamatsu) at ×40 magnification for analysis in NDP.view2 software.

### Laser capture microdissection sequencing

LCM was performed in RNase-free conditions according to a published protocol^41^. Pools of 160 cells were collected from H&E-stained frozen sections for BCs, DNs and normal-appearing neurons (NNs) based on their morphology, as previously detailed^12^. DNs and BCs were identified as cytomegalic cells (largest soma diameter >20 μm), with (DNs) or without (BCs) Nissl substance cytoplasmic aggregates. NNs with oval shape, largest soma diameter between 10 μm and 20 μm and no Nissl substance aggregates were collected from perilesional regions, distant from cytomegalic cells. For DN and BC selection, we selected 5 specimens with optimal frozen section quality and ≥50 DNs or BCs per section. For patients pt2 and pt4, duplicate DN pools were collected from distinct cortical gyri. Microdissected cells were collected and RNA was extracted within 2 h of tissue staining using the RNA Tissue XS kit (Macherey-Nagel). RNA samples were stored at −80 °C. SmartSeq libraries were generated using the Nextera XT DNA Library Kit (for low input) and sequenced on MiSeq Standard v.2 (2× 150 bp). Transcript expression quantification was performed using Salmon v.1 (Genecode v.36 reference) and VST (variance stabilizing transformation) normalization. Samtools mpileup (v.0.1.9) was used to extract the sequencing coverage at the mutation sites in each sample. Mutated reads were manually inspected using the Integrative Genome Viewer (IGV, v.2.16.2) at each mutation site to assess the presence of the somatic mutation.

### Dataset integration and cell-type assignment

To ascertain the cell-type identity of the sequenced nuclei (snRNA-seq) and of the LCM–seq samples, we utilized control cells from a previously published prefrontal cortex dataset as a reference (here referred to as V19)^22^. Specifically, we randomly selected *n* = 350 V19 control nuclei per cell type from individuals aged between 4 years and 19 years, aligning with the age range of our cohort (5538_PFC_Nova, 5387_BA9, 5408_PFC_Nova, 5936_PFC_Nova, 5893_PFC, 5879_PFC_Nova, 5976_BA9 and 4341_BA46). To mitigate batch effects between V19 individuals, we executed the Seurat (v.2) single-cell integration pipeline. Next, we integrated each in-house dataset on to V19 using a common set of 2,000 variable genes across datasets. SnRNA-seq and LCM samples were sequentially integrated with the V19 reference to optimize parameter tuning based on sample sizes. Finally, a common scaled data space was assembled across integrations (intra-V19, snRNA-seq-V19, LCM-V19) and, on UMAP 2D embedding, we trained a crossvalidated (10-fold) 200-nearest neighbors’ classifier on V19 cell coordinates, achieving an 87% accuracy to predict the maximum likelihood cell type of in-house integrated samples. Cell-type annotation was slightly adjusted based on a recent publication^42^: the V19 cluster annotations ‘NRGN-I’, ‘NRGN-II’ and ‘Mat.Neu’ were here categorized under ‘GluNs’, whereas ‘Ast-PP’ and ‘Ast-FB’ were combined and labeled as ‘astrocytes’.

### UMAP plots and cell-type proportions

To illustrate the distribution of nuclei based on age and to depict single individual UMAPs, 500 random nuclei per control or patient from our dataset were plotted alongside 500 random nuclei from the control reference dataset (V19; by age: 4 years, *n* = 29; 6 years *n* = 53; 12 years, *n* = 147; 13 years, *n* = 78; 14 years, *n* = 46; 15 years, *n* = 38; and 19 years, *n* = 109). To analyze nuclei distribution according to dysplasia type (that is, control, focal or hemispherical dysplasia), disease group (that is, patients versus controls) or predicted cell types, we randomly selected equal numbers of nuclei per individual, totaling 3,000 nuclei per phenotype (controls: 750 nuclei per individual, *n* = 4; hemispherical: 1,000 nuclei per individual, *n* = 3; focal: 430 nuclei per individual, *n* = 7; and patients: 300 nuclei per individual, *n* = 10). LCM samples (8 DN, 4 NN and 2 BC pools) were plotted with control nuclei (750 from each in-house control and 750 from the V19 dataset; total *n* = 3,000).

### PacBio long-read sequencing

Targeted long-read sequencing was performed on 10× single-nucleus complementary DNA from patients pt8 and pt10. Single-nucleus cDNA (50 ng) from 10x Genomics libraries underwent initial amplification (four to six PCR cycles) using NEBNext Ultra II Q5 Master Mix (New England Biolabs, cat. no. M0544L) following the manufacturer’s recommended procedure. A customized probe panel (Twist Biosciences) targeting 12 mTOR pathway genes (*PIK3CA*, *PTEN*, *AKT3*, *MTOR*, *TSC1*, *TSC2*, *DEPDC5*, *NPRL3*, *RHEB*, *SLC35A2*, *BRAF* and *NPRL2*) was used for hybridization (850 ng of purified cDNA, 16 h). Post-capture PCR amplification (16 cycles, 69 °C annealing) was conducted using NEBNext Ultra II Q5 Master Mix. SMRTbell libraries were prepared using the SMRTbell Express Template Prep Kit 2.0 following PacBio’s protocol. Circular consensus sequences with ~99.9% precision were generated using ccs software and demultiplexed (lima v.2.7.1). Sequences were analyzed using a customized pipeline to recover the barcodes of Mut. and Ref. nuclei: (1) reads were aligned to the GRCh38 human genome reference (minimap2 v.2.24); (2) patient-specific variants were identified (GenomicAlignments v.1.42.0 R module); and (3) reads were demultiplexed for 10× barcodes using the script seal.sh (BBtools suite v.38.96) (only barcodes with 100% identity were recovered).

### SnRNA-seq nuclei genotyping

To detect variants in single nuclei and overcome coverage limitations of standard snRNA-seq data, we adapted a previously reported approach^43^, combining high-throughput 10x Genomics snRNA-seq with targeted PacBio long-read sequencing of barcoded transcripts, which produces highly accurate results^44^. The cb_sniffer (v.1.0) was used to provide a list of nuclei barcodes for which the mutation site was covered in snRNA-seq datasets. We classified nuclei as Mut. (at least one mutated read) or Ref. (only reads with reference alleles). Given that each cell is diploid and read depth is limited in snRNA-seq data, cells classified as Ref. may still carry the mutation but lack coverage of the mutant allele. All mutation sites were manually verified using IGV (v.2.16.2) to ensure high-quality variant calling (that is, removing ambiguously mapped or duplicate reads, confirming the absence of nearby variants that suggest sequencing error). Total coverage and coverage per nucleus were extracted with Samtools mpileup (v.0.1.9). Control snRNA-seq datasets were used to calculate the false-positive (FP) rate at mutation sites using Samtools mpileup (v.0.1.9) for the variant calling. The FP rate was determined as: ((no. of observed mutated reads − no. of expected mutated reads)/no. of total reads).

### FANS

We isolated nuclei from ~100 mg of frozen cortical tissue from *n* = 7 FCDII cases: *n* = 2 with the *MTOR* p.A1459D somatic variant (pt4 and pt13); *n* = 2 with the *MTOR* p.S2215F somatic variant (pt11–12); *n* = 2 with the *PIK3CA* p.E545K somatic variant (pt14–15); and *n* = 1 with the *RHEB* p.Y35L somatic variant (pt9) (details of individuals included in each experiment are in Supplementary Tables 1 and 2). Isolation of nuclei was performed as for snRNA-seq, except for a fixation step in 1% paraformaldehyde (PFA) added after the first homogenization. Nuclei were resuspended in staining buffer (2% bovine serum albumin, 1 mM EDTA and phosphate-buffered saline) and immunostained overnight at 4 °C with the following primary antibodies: conjugated anti-NEUN-PE for neurons (1:1,000, Milli-Mark, cat. no. FCMAB317PE)^45^, conjugated anti-PU.1-AF647 for microglia (1:100, Cell Signaling Technology, cat. no. 2240S conjugate)^46,47^, unconjugated anti-OLIG2 for oligodendrocytes (1:500, Abcam, cat. no. ab109186)^30^, conjugated anti-PAX6-APC for astrocytes (1:1,000, Novus Biologicals, cat. no. NBP2-34705APC)^48^ and rabbit unconjugated anti-TBR1 for excitatory neurons (1:1,000, Abcam, cat. no. ab31940)^30^. Nuclei were then incubated for 1 h at 4 °C with DAPI (1:1,000, Thermo Fisher Scientific, cat. no. D1306); OLIG2^+^ nuclei were incubated with the secondary antibody donkey anti-rabbit Alexa Fluor-647 (1:1,000, Invitrogen, cat. no. A13573), TBR1^+^ nuclei were incubated with secondary antibody donkey anti-rabbit-phycoerythrin/Atto594 (1:1,000, Novus Biologicals, cat. no. NBP1-75286PEATT594). Stained nuclei were filtered through a 40-μm strainer and sorted using MoFlo Astrios (Beckman Coulter) with a 70-μm nozzle. Gating was performed based on DAPI and fluorescent tags using nuclei from control frozen brain tissue, processed together with patients’ samples. DNA was extracted using a NucleoSpin Tissue kit (Macherey-Nagel, cat. nos. 740952.250 or 740901.250).

### Droplet digital PCR

Droplet digital PCR (ddPCR) (QX200 system, BioRad Laboratories) was performed as previously described^49^ using commercial assays (FAM + HEX) from BioRad: *MTOR* p.S2215F, *MTOR* p.A1459D and *PIK3CA* p.E545K. Data were analyzed with the QX Manager (v.1.2) software. DNA samples from bulk brain tissue of mutation-positive patients were used as positive controls and DNA from blood samples of 13 nonepileptic individuals as negative controls. Equivalent DNA amounts (1–4 ng) were used across all reactions with the same ddPCR probe (Supplementary Table 8). Technical replicates (range 2–4) were performed when sufficient DNA was available. The VAF for each sample was calculated as the average fractional abundance across available replicates. The limit of blank (LOB) and limit of detection (LOD) were calculated for each probe as previously described^49^ and used to define mutation-positive and mutation-negative samples (no. of FAM^+^ droplets above and below the LOD, respectively).

### Targeted amplicon sequencing

*RHEB* p.Y35L variant (pt9) detection used deep targeted amplicon sequencing (TAS; 14,752× mean coverage, range 1,175× to 25,312×), because no commercial ddPCR probes were available. Genomic DNA extracted from pt9’s bulk brain resected tissues was used as a mutation-positive control and blood samples from five healthy controls to account for possible sequencing artifacts as mutation-negative controls. PCR amplicons (243 bp) were obtained from 10 ng of genomic DNA from mutation-positive and mutation-negative control samples and the entire extraction volume for the FANS-enriched DNA samples. Libraries were sequenced on MiSeq (2× 250 bp). Variants were called following alignment to GRCh38 using a GATK pipeline (GenomeAnalysisTK-3.8-1-0) and SAMtools (v.0.1.9). To ensure high-quality variant calls, all sequencing results were manually verified using IGV (v.2.16.2).

### Visium spatial transcriptomics

Frozen brain sections (10 µm) from patients pt2, pt5 and pt9 were processed using a standard Visium protocol (55-µm spot diameter). Libraries were sequenced on a NovaSeq 6000 SP (2× 50 bp), analyzed using Space Ranger (v.2.0.0) and aligned with the ‘refdata-gex-GRCh38-2020-A’ reference genome. Tissue-overlapping spots were manually selected using Loupe Browser (v.6), with DN and BC spots specifically annotated. Quality filtering in Seurat (v.4.1.1) removed spots with <200 genes, <500 reads, >40% mitochondrial genes, >20% hemoglobin genes or >5% ribosomal genes, and mitochondrial DNA-encoded genes were removed. Data were normalized with Seurat’s SCTransform. Principal component analysis (PCA) reduction was performed using the marker genes from a previously published control Visium dataset^50^. UMAP reduction and clustering were conducted with clustering resolutions of 0.4 (pt2 and pt9) and 0.2 (pt5). Spatial clusters were annotated based on predominant cell-type markers identified by FindAllMarkers function (Seurat). When more than one cluster was predominantly characterized by the same cell type, the top expressed marker was included in the annotation for their distinction. Cell-type prediction and deconvolution were performed with CytoSPACE (v.1.0.1)^51^ to estimate the number of cells per spot. The ‘AddModuleScore’ Seurat function was used to evaluate the expression pattern of cortical layer markers per spot, based on the gene lists provided by a previously published control Visium dataset^50^.

### Identification of DN dysregulated genes

We intersected the dysregulated genes identified with snRNA-seq in GluN mutated nuclei, LCM–seq DN samples and Visium DN-containing spots (DN up- or downregulated genes), followed by GO analysis of DN upregulated genes.

### MERSCOPE spatial transcriptomics

We designed a customized panel of 140 genes, comprising 67 new candidate biomarkers of DNs (*n* = 30) and BCs (*n* = 37) selected across snRNA-seq, LCM–seq and Visium data. The panel also included 16 cell-type markers, 7 mTOR pathway genes, 9 senescence-associated genes^52^ and 41 DN or BC putative markers from the literature. Frozen cortical sections (10 μm thick) from pt5 and pt9 were processed following the standard Vizgen protocol. Sections were stained with DAPI to mark nuclei and antibodies against NEUN and pS6^240/244^ to distinguish DNs (NEUN^+^/pS6^+^ cells) and BCs (pS6^+^/NEUN^−^ cells). Regions of interest (ROIs), manually selected on the MERSCOPE visualizer around single DNs (*n* = 100, 50 from each patient), BCs (*n* = 100, 50 from each patient), astrocytes (*n* = 50 from pt5) and GluNs (*n* = 100, 50 from each patient) were based on NEUN/pS6 immunoreactivity and expression of known markers (*NEFM* for DNs, *VIM* for BCs, *AQP4* for astrocytes and *NRGN* for GluNs). The gene expression matrix and coordinates for each ROI were exported and processed into a Seurat (v.4) object for analysis of raw gene counts for the 140 genes: (1) SCTransform gene count normalization to the total expression; (2) highly variable gene detection and PCA; (3) graph-based clustering (with the ten first PCs and a clustering resolution of 0.5); and (4) UMAP calculation. Heatmaps of SCT-normalized gene expression data for known and new markers were generated using the ComplexHeatmap (v.2.16) package in R.

### Electron microscopy

Electron microscopy (EM) was performed in patients pt4, pt5 and pt7. After surgical removal, fresh tissue samples (5 mm^3^) from pt4 and pt5 were incubated 1 h at room temperature (RT) in a fixative solution (2% glutaraldehyde, 2% PFA and 2 mM CaCl_2_ in 0.1 M sodium cacodylate buffer, pH 7.4). For pt7, a 40-μm-thick cryosection was fixed. Samples were post-fixed for 1 h at RT in 1% osmium tetroxide and contrasted with 2% uranyl acetate. Pieces of gay matter were dissected, progressively dehydrated in ethanol solutions and acetone and embedded in Epon resin (polymerization for 48 h at 56 °C). Semi-thin sections (0.5 μm thick) were stained with 1% toluidine blue/1% borax and ultra-thin sections (70 nm thick) were contrasted with Reynold’s lead citrate. Images were acquired using a Hitachi HT7700 electron microscope (70 kV) equipped with an AMT41B camera.

### Statistical analyses

All statistics and graphs were generated in R (ggplot2 package v.3.4.3). No statistical methods were used to predetermine sample sizes, but our sample sizes are similar to those reported in previous publications^14^. Histological stainings were performed blind to specific genetic etiologies, whereas all other analyses were not:

1) Differential gene expression analyses: analyses were performed using the FindMarkers and FindAllMarkers functions (Seurat R package v.5.0.3) with default parameters and the Wilcoxon’s rank-sum test. (i) Patients versus controls included focal lesion patients (pt2–3, pt5–8 and pt10) for which resected tissue is within the epileptogenic focus compared with tissue resected after hemispherotomy. Gene expression was compared between focal patients and age-matched control nuclei (aged 2–19 years), using the same number of randomly selected nuclei per individual and phenotype: astrocytes (*n* = 350 nuclei per individual); oligodendrocytes (*n* = 200 nuclei per control, *n* = 65 nuclei per patient); microglia (*n* = 200 nuclei per control, *n* = 300 nuclei per patient); GluNs (*n* = 350 nuclei per control, *n* = 80 nuclei per patient); IN-CGEs (*n* = 350 nuclei per individual); IN-MGEs (*n* = 350 nuclei per individual); GluL2/3 (*n* = 350 nuclei per control, *n* = 150 nuclei per patient); GluL4 (*n* = 350 nuclei per control, *n* = 200 nuclei per patient); and GluL5/6 (*n* = 350 nuclei per control, *n* = 250 nuclei per patient). GO analysis was performed using the gseGO function (clusterProfiler R package)^53^ on genes expressed in >25% of cells (*P*_adj_ < 0.05, absolute log_2_(fold-change) (log_2_(FC)) > 0.4), as previously described for postoperative tissue snRNA-seq analysis^48^. (ii) Mut. versus Ref. compared nuclei from *RHEB*-mutated patients’ (pt9 or pt10) nuclei: GluNs: *n* = 29 Mut. (pt9: 8; pt10: 21) and *n* = 92 Ref. nuclei (pt9: 7; pt10: 85); GluL2-6: *n* = 22 Mut. (pt9: 12; pt10: 10) and *n* = 127 Ref. nuclei (pt9: 9; pt10: 118); astrocytes: *n* = 17 Mut. (pt9: 1; pt10: 16) and *n* = 50 Ref. nuclei (pt10 only); and oligodendrocytes (pt10 only): *n* = 17 Mut. and *n* = 101 Ref. nuclei. GO analysis was performed using the gseGO function on genes with log_2_(FC) > 0.3 (more stringent cutoff values did not allow pathway enrichment analyses). (iii) Patient Ref. versus controls compared gene expression for GluN Ref. nuclei from pt9 and pt10 (>10 mutated cells) with age-matched control nuclei from ct1 and ct2. GO analysis was performed using the gseGO function on genes expressed in >25% (absolute log_2_(FC) > 0.3).

2) Correlation analysis was performed using the cor.test(x, y) function in R with Pearson’s correlation coefficient to assess relationships between DEG counts and cell or gene numbers per cell type.

3) The mutation distribution across cell types was analyzed using a *χ*^2^ test on the observed data. To assess significance, we generated 10,000 random cell count distributions under the assumption of equal probabilities for each cell type using a multinomial distribution. A *χ*^2^ statistic was computed for each random distribution. Statistical significance was assessed by comparing the observed statistic against this permuted distribution (*P* < 0.05 threshold). The sensitivity of this approach was validated through power analysis using simulations with predefined effect sizes.

4) Validation of mutation-driven transcriptional changes: we compared the dysregulated genes between Mut. and Ref. GluNs or astrocytes (referred to as ‘observed’ changes) with gene expression differences from randomly sampled GluNs or astrocyte nuclei. We performed 50 random selections of GluNs (*n* = 29) or astrocytes (*n* = 14) and compared them with the remaining population of GluNs and astrocytes from both Mut. and Ref. nuclei. For each iteration, dysregulated genes were identified using the same threshold of absolute log_2_(FC) > 0.3 and compared with the observed dysregulated genes using the Jaccard similarity index (1 indicates 100% overlap). Statistical significance was determined by comparing the distribution of Jaccard indices against observed gene sets.

5) Predictive modeling of mutated cell identity: linear regression models were built using the bmrm R (v.4.1) package to predict mutated cell identity based on ‘observed’ or ‘random’ dysregulated genes. Models were tested through crossvalidation techniques, and their specificity and sensitivity were assessed via the area under the receiver operating characteristic curve. These models were additionally used to estimate the false-negative rate among Ref. nuclei (Extended Data Fig. 5b), because Ref. nuclei with transcriptional profiles similar to Mut. nuclei could represent undetected mutation-carrying cells.

6) Selection of top marker genes in Visium spatial transcriptomics: the top five markers for DNs, BCs and other spots were identified using FindAllMarkers (Seurat v.4) and ordered by decreasing log_2_(FC), using default parameters except: logfc.threshold = 0.25 and min.pct = 0.1. The adjusted *P* (*P*_adj_) value significance threshold was set at <0.05 (Bonferroni’s correction across all genes).

7) Selection of top marker genes in MERSCOPE spatial transcriptomics: the top ten DN or BC markers in the merged Seurat object were identified using FindAllMarkers (Seurat v.4) and ordered by decreasing log_2_(FC), using default parameters except: logfc.threshold = 1 and min.pct = 0.5. The *P*_adj_ value significance threshold was set at <0.05 (Bonferroni’s correction across all genes).

8) VDAC1 immunofluorescence quantification: whole-slide scans (CZI format) were analyzed using QuPath (v.0.5.1), with pixel thresholding to measure the percentage of VDAC1^+^/pS6^+^ pixels in 0.25-mm^2^ ROIs. For patient pt2, we compared cortical regions with and without DNs (4 ROIs per region). For patients pt5–8, we compared regions containing either DNs or BCs (3 ROIs per region per patient). Two-tailed Wilcoxon’s rank-sum exact test was used to compare the percentage of VDAC1^+^ pixels between (i) DN-positive and DN-negative regions in pt2 and (ii) DN-positive and BC-positive regions in pt5–8. Significance was determined by the exact *P* values obtained from the test.

### Reporting summary

Further information on research design is available in the Nature Portfolio Reporting Summary linked to this article.

## Online content

Any methods, additional references, Nature Portfolio reporting summaries, source data, extended data, supplementary information, acknowledgements, peer review information; details of author contributions and competing interests; and statements of data and code availability are available at 10.1038/s41593-025-01936-z.

## Supplementary information

Reporting Summary

Supplementary Tables Supplementary Table 1. Clinical, demographic and genetic characteristics of patients with FCDII and control subjects. Somatic variants were not detected in pt3. na, not available; Low: 3–6%; Medium: 7–10%; High: 11–36%; y, years; DN, dysmorphic neurons; BC, balloon cells. Supplementary Table 2. Number of individuals included across experiments and analyses. snRNA-seq, single-nuclei RNA sequencing; LCM–seq, laser-capture microdissection coupled with sequencing; FANS, fluorescence activated nuclei sorting; ddPCR, droplet digital polymerase chain reaction; TAS, targeted amplicon sequencing; HE, hematoxylin/eosin; IHC, immunohistochemistry; FFPE, formalin-fixed paraffin embedded; DN, dysmorphic neurons; BC, balloon cells; co-IF: co-immunofluorescence; DEGs, differentially expressed genes; UMAP, uniform manifold approximation and projection. Supplementary Table 3. Quality metrics for single-nucleus RNA sequencing. avg nCount, average number of mRNA molecules per nucleus; avg nFeature, average number of unique features per nucleus; avg nUMI, average number of unique molecular identifiers per nucleus; avg nGene, average number of unique genes per nucleus; avg pct.mt, average percentage of mitochondrial genes per nucleus. Supplementary Table 4. Differential expression analysis between patients with FCDII and controls. List of genes up-/downregulated (absolute average log2 fold change (avg_log2FC) > 0.4) between patients and controls per cell type. Genes are ordered by increasing avg_log2FC. Supplementary Table 5. Distribution of genotyped nuclei across patients with FCDII. A total of *n* = 808 nuclei were genotyped. Samples number pt1 and pt3 were not included since the somatic hit was either not a point mutation or not identified by DNA sequencing. VAF, variant allele frequency, Glu, glutamatergic; N, neurons; L, layer; CC, cortico-cortical projection neurons; IN, interneurons; Astro, astrocytes; Oligo, oligodendrocytes, OPC, oligodendrocyte precursor cells; Micro, microglia; Endo, endothelial cells. Supplementary Table 6. Mutation site coverage in control samples from snRNA-seq data. Total sequencing coverage was obtained from IGV visualization of Cell Ranger output BAM files. mut = number of mutated reads per sample; total = number of total reads per sample. Supplementary Table 7. Cell type-specific sequencing coverage from snRNA-seq and PacBio data. Sequencing coverage per genotyped nucleus barcode is expressed as number of reads on the mutation site; when variable across nuclei, the mean coverage and range is reported. ‘-’ indicates no coverage. Glu, glutamatergic; N, neurons; L, layer; CC, cortico-cortical projection neurons; IN, interneurons; Astro, astrocytes; Oligo, oligodendrocytes, OPC, oligodendrocyte precursor cells; Micro, microglia; Endo, endothelial cells. Supplementary Table 8. Genotyping results from sorted nuclei populations by ddPCR and TAS. Genotyping was performed on DNA from nuclei sorted by fluorescence-activated nuclei sorting (FANS) using droplet digital PCR (ddPCR) or targeted amplicon sequencing (TAS). One tissue sample per patient was processed for nuclei isolation and divided for immunostaining. Technical ddPCR replicates were performed when sufficient DNA was available. The FAM signal indicates the mutated allele, while the HEX signal indicates the wild-type allele. Supplementary Table 9. DN and BC counting in FCDII specimens. For all patient, one tissue section was immunostained for SMI311 to mark dysmorphic neurons (DN) and counterstained with DAPI for nuclei count. For patients pt5–7 and pt10, one tissue section was immunostained for VIM to mark balloon cells (BC) and counterstained with DAPI for nuclei count. na, not available. Supplementary Table 10. Dysregulated genes in mutation detected (Mut.) vs. reference detected (Ref.) nuclei from patients with FCDII. List of genes with increased/decreased expression (absolute average log2 fold change (avg_log2FC) > 0.3) in Mut. nuclei compared to Ref. nuclei per cell type. Genes are ordered by increasing avg_log2FC. Supplementary Table 11. Dysregulated genes in FCDII reference detected (Ref.) nuclei vs. controls (Non-cell-autonomous transcriptional changes). List of genes with increased/decreased expression (absolute average log2 fold change (avg_log2FC) > 0.4) in Ref. nuclei compared to control nuclei per cell type. Genes are ordered by increasing avg_log2FC. Supplementary Table 12. LCM–seq gene expression matrix (VST-normalized). Pools of 160 cells were isolated by LCM for RNA extraction and sequencing. Dysmorphic neurons (DN) were isolated from samples number pt1, pt2 (*n* = 2 pools), pt3, pt4 (*n* = 2 pools), pt5, pt9; balloon cells (BC) were isolated from samples number pt5 and pt9; normal neurons (NN) were isolated from samples number pt4 (*n* = 2), pt7, pt8. Supplementary Table 13. MERSCOPE gene panel. List of genes included in the MERSCOPE panel grouped by category. Supplementary Table 14. List of 57 genes upregulated in DN (Convergent DN-enriched gene signature). Genes upregulated in Mut. compared to Ref. GluN from patients with FCDII, also showing expression levels higher in Visium spots containing dysmorphic neurons (DN) and DN-LCM–seq samples (compared to the other Visium spots or LCM–seq samples). Average log2 fold change (avg_log2FC) values are reported. Genes with ontologies related to metabolism and mitochondria are in red.

## Data Availability

Raw snRNA-seq patient data are deposited at the European Genome-Phenome Archive (EGA; https://ega-archive.org, accession nos. EGAD50000001403 and EGAS50000000964) accession study title ‘10X snRNA-seq data from human FCDII postoperative brain tissues with mTOR pathway mutations’. Data are available to academic researchers under controlled access as a result of the sensitive nature of the sequencing data, by contacting the appropriate Data Access Committee (EGA accession no. EGAC50000000546). Raw and processed Visium, MERSCOPE, gene panel sequencing data and histological scans are available to academic researchers upon request to the corresponding author, subject to a signed data transfer agreement. The GRCh38 human genome references used for transcriptomic and genomic analyses were retrieved from the 10x Genomics (https://www.10xgenomics.com) and GATK (https://gatk.broadinstitute.org) websites. Control snRNA-seq raw data from Velmeshev et al.^22^ were made available by the authors of the original publication through the Sequence Read Archive, accession no. PRJNA434002.

## Code availability

Standard codes were used in this study and will be made available by the authors upon request.
