# Supplementary material for: Single-cell genotyping and transcriptomic profiling of mosaic focal cortical dysplasia
Source: Nat Neurosci. 2025 Apr 30;28(5):964–72. doi: 10.1038/s41593-025-01936-z (PMC12081288; doi:10.1038/s41593-025-01936-z)
Supplement: Supplementary file 1 — Reporting Summary [file 41593_2025_1936_MOESM1_ESM.pdf]

nature portfolio

Corresponding author(s): Stéphanie Baulac

Last updated by author(s): Feb 6, 2025

Reporting Summary

Nature Portfolio wishes to improve the reproducibility of the work that we publish. This form provides structure for consistency and transparency in reporting. For further information on Nature Portfolio policies, see our Editorial Policies and the Editorial Policy Checklist.

Statistics

For all statistical analyses, confirm that the following items are present in the figure legend, table legend, main text, or Methods section.

n/a	Confirmed	
☐	☒	The exact sample size (*n*) for each experimental group/condition, given as a discrete number and unit of measurement
☐	☒	A statement on whether measurements were taken from distinct samples or whether the same sample was measured repeatedly
☐	☒	The statistical test(s) used AND whether they are one- or two-sided<br>*Only common tests should be described solely by name; describe more complex techniques in the Methods section.*
☒	☐	A description of all covariates tested
☒	☐	A description of any assumptions or corrections, such as tests of normality and adjustment for multiple comparisons
☐	☒	A full description of the statistical parameters including central tendency (e.g. means) or other basic estimates (e.g. regression coefficient) AND variation (e.g. standard deviation) or associated estimates of uncertainty (e.g. confidence intervals)
☐	☒	For null hypothesis testing, the test statistic (e.g. *F*, *t*, *r*) with confidence intervals, effect sizes, degrees of freedom and *P* value noted<br>*Give P values as exact values whenever suitable.*
☒	☐	For Bayesian analysis, information on the choice of priors and Markov chain Monte Carlo settings
☒	☐	For hierarchical and complex designs, identification of the appropriate level for tests and full reporting of outcomes
☒	☐	Estimates of effect sizes (e.g. Cohen's *d*, Pearson's *r*), indicating how they were calculated

Our web collection on statistics for biologists contains articles on many of the points above.

Software and code

Policy information about availability of computer code

Data collection

- 10X snRNA-seq and Visium: fastq files were obtained from an Illumina Novaseq 6000 sequencing platform;
- Control 10X snRNA-seq from Velmeshev et al 2019: raw data downloaded from the Sequence Read Archive, accession number PRJNA434002 for samples: 5538_PFC_Nova, 5387_BA9, 13 5408_PFC_Nova, 5936_PFC_Nova, 5893_PFC, 5879_PFC_Nova, 5976_BA9, 4341_BA46.
- LCM-seq: fastq files were obtained from an Illumina MiSeq sequencing platform;
- MERSCOPE data were acquired using the Vizgen MERSCOPE platform;
- PacBio long-read: raw data from a PacBio Sequel sequencer
- Histological slides were scanned with a Nanozoomer scanner (Hamamatsu) and an Axioscan (Zeiss).
- ddPCR results were collected via the QX200 system (Bio-Rad Laboratories)
- TAS fastq files were obtained from an Illumina MiSeq sequencing platform.

Data analysis

Softwares used:
- 10X snRNA-seq: Cell Ranger v3 (reads aligned on the GRCh38 human reference genome including intronic regions), R packages: DropletUtils v1.4.3, clusterProfiler v4.5.3, Seurat v2, ggplot2 v3.4.3 and other basic R packages. Integrative Genome Viewer (IGV) v2.16.2, samtools mpileup v0.1.9, bmrm R package v4.1, cb_sniffer v1.0
- LCM-seq: Salmon v1, IGV v2.16.2, R packages: Seurat v2, ggplot2 v3.4.3 and other basic R packages.
- 10X Visium: Space Ranger v2 (reads aligned on "refdata-gex-GRCh38-2020-A"), Loupe Browser v6, CytoSPACE v.1.0.1, R packages: Seurat v4, ggplot2 v3.4.3, ComplexHeatmap v2.16.
- MERSCOPE: MERSCOPE Visualizer v2, R packages: Seurat v4, ggplot2 v3.4.3, ComplexHeatmap v2.16.
- PacBio long-read: ccs v7.0.0, lima v2.7.1, minimap2 v2.24 (hg38 human genome reference), BBtools v38.96 (script seal.sh), IGV v2, R packages: GenomicAlignements v1.42.0.

- Histological images: NDP.view2, Zen 2.3 Blue, QuPath v.0.3.2 and v.0.5.1
- ddPCR: QX Manager v1.2 software (Bio-Rad Laboratories)
- TAS: GATK pipeline (GenomeAnalysisTK-3.8-1-0), SAMtools v0.1.9, IGV v2.16.2.

For manuscripts utilizing custom algorithms or software that are central to the research but not yet described in published literature, software must be made available to editors and reviewers. We strongly encourage code deposition in a community repository (e.g. GitHub). See the Nature Portfolio guidelines for submitting code & software for further information.

Data

Policy information about availability of data

All manuscripts must include a data availability statement. This statement should provide the following information, where applicable:

- Accession codes, unique identifiers, or web links for publicly available datasets
- A description of any restrictions on data availability
- For clinical datasets or third party data, please ensure that the statement adheres to our policy

Raw snRNA-seq patient data are deposited at the European Genome-Phenome Archive (EGA; https://ega-archive.org), accessionstudy title "10X snRNA-seq data from human FCDII postoperative brain tissues with mTOR pathway mutations". Data are available to academic researchers under controlled access due to the sensitive nature of the sequencing data, by contacting the appropriate Data Access Committee (EGAC50000000546). Raw and processed Visium, MERSCOPE, gene panel sequencing data and histological scans are available to academic researchers upon request to the corresponding author, subject to a signed Data Transfer Agreement (DTA). The GRCh38 human genome references used for transcriptomic and genomic analyses were retrieved from the 10X Genomics (https://www.10xgenomics.com/) and GATK (https://gatk.broadinstitute.org) websites. Control snRNA- raw sequencing data from Velmeshev et al. (2019) are available through the Sequence Read Archive, accession number PRJNA434002.

Research involving human participants, their data, or biological material

Policy information about studies with human participants or human data. See also policy information about sex, gender (identity/presentation), and sexual orientation and race, ethnicity and racism.

Reporting on sex and gender	Sex of the patients and controls included in the study was obtained via self-reporting. Sex was not used for gender-based analyses since no phenotypic difference is reported between male and female patients with FCDII.
Reporting on race, ethnicity, or other socially relevant groupings	No information on race, ethnicity or other socially relevant groupings is provided in the study.
Population characteristics	The study included 15 patients (age range: 3 months - 16 years) with drug-resistant focal epilepsy and 3 postmortem cortical samples from non-epileptic age-matched controls. All patients had either somatic mutations in MTOR, RHEB or PIK3CA genes, or germline and somatic variants in DEPDC5. All patients received a neuropathological diagnosis of FCDII after neurosurgery.
Recruitment	FCDII patients were recruited by the epilepsy surgery department at the Rothschild Foundation Hospital (Paris, France) between 2016 and 2021. Inclusion criteria were: (i) neuropathological diagnosis of FCDII according to the ILAE classification, (ii) genetic testing revealing somatic mutations in mTOR pathway genes, and (iii) availability of high-quality surgical tissue samples (confirmed by histological examination, DNA and RNA integrity assessment)..<br><br>Control material was obtained from three age-matched postmortem frontal lobe tissues of individuals without a history of seizures or other neurological diseases. The mean age at death was 4.1 years (range 2 months to 10 years), with a mean postmortem interval of 7 hours before brain tissue collection (range 6.5h-7.5h). No compensation was provided.
Ethics oversight	Patients recruited at the Rothschild Foundation Hospital (Paris, France) provided written informed consent to the study approved by the Île-de-France II Committee of Protection of Persons (ID-RCB/EUDRACT-2015-A00671-48). The control tissues collection and usage adhered to the principles outlined in the Declaration of Helsinki and the Amsterdam UMC Research Code provided by the Medical Ethics Committee (authorization N° W21_295 # 21.326).

Note that full information on the approval of the study protocol must also be provided in the manuscript.

Field-specific reporting

Please select the one below that is the best fit for your research. If you are not sure, read the appropriate sections before making your selection.

☒ Life sciences ☐ Behavioural & social sciences ☐ Ecological, evolutionary & environmental sciences

For a reference copy of the document with all sections, see nature.com/documents/nr-reporting-summary-flat.pdf

Life sciences study design

All studies must disclose on these points even when the disclosure is negative.

Sample size	No statistical methods were used to pre-determine sample sizes as sample size is limited by the availability of human brain material. We

Sample size	collected resected tissues from as many cases as possible, and our cohort is larger than recently reported snRNA-seq studies on FCDII (PMID: 36635388 and 36631516).<br>- Long-read sequencing: n=2 patients with cDNA available after 10X snRNA-seq experiment<br>- Histological stainings: performed only in patients (no tissue available for postmortem controls), 1 slide per patient:<br>- hematoxyilin and eosin coloration: n=10,<br>- pS6-IHC: n=10,<br>- VIM-IHC: n=10,<br>- SMI311-IHC: n=10,<br>- GFAP/pS6 co-IF: n=8,<br>- IBA1/pS6 co-IF: n=5,<br>- NEUN/pS6 co-IF: n=4,<br>- NRGN/pS6/SMI311 co-IF: n=10,<br>- NRGN/pS6/VIM co-IF: n=7,<br>- OLIG2/pS6 co-IF: n=1,<br>- pS6/SMI311 co-IF: n=10,<br>- pS6/VDAC1 co-IF: n=10.<br>- LCM-seq: n=8 patients with sufficient histological quality to allow recognition of cell types and sufficient number of pathological cells to allow laser microdissection of 160 cells per cell group within a 2 hours limit timeframe.<br>- 10X Visium: n=3 patients, one for each genetic etiology;<br>- MERSCOPE: n=2 patients, chosen among the ones used for Visium and presenting both dysmorphic neurons and balloon cells.<br>- Electron microscopy: n=3 patients, performed on cases with neuropathological FCDII suspicion based on MRI findings and sufficient tissue available.<br>- FANS followed by ddPCR/TAS genotyping: n=7 patients, selected based on tissue availability and somatic mutation identified.
Data exclusions	No data were excluded from the analysis
Replication	Replication was not performed for each snRNAseq, Visium, MERSCOPE and immunostaining experiments due to human tissue limitations.<br><br>FANS-sorting and ddPCR technical replicates (range 2-4) were included for samples/cell-sorted populations with sufficient DNA available for the analysis
Randomization	Randomization was not performed: this is a descriptive study and individuals were included in patients or controls groups based on their epileptic phenotype, neuropathological diagnosis and genetic etiology.
Blinding	Histological quantification was performed only on patients (no available tissue for controls), the experimenter was blind to the specific genetic etiology. No blinding was applied for all other analyses since they were performed using automatic bioinformatic pipelines comparing control and disease groups.

Reporting for specific materials, systems and methods

We require information from authors about some types of materials, experimental systems and methods used in many studies. Here, indicate whether each material, system or method listed is relevant to your study. If you are not sure if a list item applies to your research, read the appropriate section before selecting a response.

Materials & experimental systems

n/a	Involved in the study	
☐	☒	Antibodies
☒	☐	Eukaryotic cell lines
☒	☐	Palaeontology and archaeology
☒	☐	Animals and other organisms
☐	☒	Clinical data
☒	☐	Dual use research of concern
☒	☐	Plants

Methods

n/a	Involved in the study	
☒	☐	ChIP-seq
☐	☒	Flow cytometry
☒	☐	MRI-based neuroimaging

Antibodies

Antibodies used	<Primary antibodies><br>1. VIM: Vimentin, Clone V9, 1:200 for IHC or 1:100 for IF, Dako, #M0725, mouse<br>2. SMI311: Anti-Neurofilament Marker, 1:200 for IHC or 1:500 for IF, BioLegend, #837801, mouse, lot B284375<br>3. pS6-240/244: Phospho-S6 Ribosomal Protein (Ser240/244), D68F8, 1:2000 for IHC or 1:1000 for IF, Cell Signaling, #5364, rabbit, lot 8<br>4. GFAP: 1:200 for IF, Thermo Fisher, #MA5-15086, mouse, S.880.0<br>5. NRGN: 1:50 for IF, Thermo Fisher, #PA5-19209, goat, lot S2C2<br>6. OLIG2: 1:100 for IF, R&D Systems, #AF2418, goat, lot UPA0922051<br>7. IBA1: 1:500 for IF, Abcam, #ab5076, goat, lot GR3460639-1<br>8. NEUN: 1:500 for IF, Millipore, #MAB377, mouse, A60, lots 2639366, 3104227 and 3832727<br>9. VDAC1: 1:500 for IF, Abcam, #ab16814, mouse, lot 232975<br><br><Secondary antibodies for IF>

10. donkey anti-mouse Alexa 555, 1:1000, Thermo Fisher #A31570, lot 2045336
11. donkey anti-rabbit Alexa 647, 1:1000, Thermo Fisher #A31573, lot 2284672
12. donkey anti-rabbit Alexa 488, 1:1000, Thermo Fisher #A21206, 2072687

<Secondary antibodies for IHC>
13. Anti-mouse, biotinylated , 1:250, Vector laboratory, #BA-2000, lot ZH0412
14. Anti-rabbit, biotinylated , 1:250, Vector laboratory #BA-1100, lot ZH0421

<Primary antibodies used for FANS>
15. Anti-NEUN-PE, 1:1000, conjugated, Milli-Mark, FCMAB317PE
16. anti-PU.1-AF647, 1:100, conjugated, Cell Signaling Technology, 2240S
17. anti-OLIG2, 1:500, Abcam, ab109186
18. anti-PAX6-APC, 1:1000, conjugated, Novus Biologicals, NBP2-34705APC
19. anti-TBR1, 1:1000, Abcam, ab31940

<Secondary antibodies for FANS>
11. donkey anti-rabbit Alexa 647, 1:1000, Thermo Fisher #A31573, lot 2284672
20. donkey anti-Rabbit-PE/Atto594, 1:1000, Novus biologicals, NBP1-75286PEATT594

Validation

<Primary antibodies>
1. VIM: monoclonal mouse antibody, raised against Vimentin (Pig); cited in 606 publications (CiteAb).
2. SMI311: Anti-Neurofilament Marker (pan-neuronal, cocktail), each lot of this antibody is quality control tested by formalin-fixed paraffin-embedded immunohistochemical staining (Supplier website).
3. pS6 detects endogenous levels of ribosomal protein S6 only when phosphorylated at Ser240 and Ser244; Product Citations: 827; Cell Signaling Technologies validate all CST® antibodies, assay kits, and reagents to ensure optimal performance in the approved applications shown on the product web pages (Supplier website).
4. GFAP: Native GFAP purified from pig spinal cord; verified by relative expression to ensure that the antibody binds to the antigen stated. RRID: AB_10981734 (Supplier website). cited in 11 publications (CiteAb)
5. NRGN: RRID: AB_10987468 (Supplier website).
6. OLIG2: polyclonal goat antibody, cited in 247 publications (CiteAb).
7. IBA1: polyclonal goat antibody, validated in IHC-P, WB and tested in Human, Rat samples; cited in 771 publications (Supplier website).
8. NEUN: monoclonal mouse antibody, cited in 6728 publications (CiteAb).
9. VDAC1: monoclonal mouse antibody, used in 10.1371/journal.pbio.3002337.

<Secondary antibodies for IF>
10. donkey anti-mouse Alexa 555: polyclonal antibody, cited in 1461 publications (CiteAb).
11. donkey anti-rabbit Alexa 647: polyclonal antibody, cited in 2377 publications (CiteAb).
12. donkey anti-rabbit Alexa 488: polyclonal antibody, cited in 6784 publications (CiteAb).

<Secondary antibodies for IHC>
13. Anti-mouse, biotinylated: polyclonal horse anti-mouse antibody, cited in 1004 publications (CiteAb).
14. Anti-rabbit, biotinylated: polyclonal horse anti-rabbit antibody, cited in 223 publications (CiteAb).

<Primary antibodies used for FANS>
15. Anti-NEUN-PE, cited 48 times (CiteAb) and used in 10.1038/s41467-024-50414-w.
16. anti-PU.1-AF647, cited 11 times (CiteAb) and used in 10.3389/fnmol.2022.948456 and 10.1126/sciadv.abo4662.
17. anti-OLIG2, cited 245 times (CiteAb), and used in 10.1038/s41586-024-07292-5.
18. anti-PAX6-APC, cited 5 times (CiteAb) and used in 10.1038/s41588-023-01547-z.
19. anti-TBR1, cited 600 times (CiteAb) and used in 10.1038/s41586-024-07292-5.

<Secondary antibodies for FANS>
11. donkey anti-rabbit Alexa 647: polyclonal antibody, cited in 2377 publications (CiteAb)
20. donkey anti-rabbit-PE/Atto594: polyclonal antibody, adapted for Flow Cytometry (supplier website)

Clinical data

Policy information about clinical studies
All manuscripts should comply with the ICMJE guidelines for publication of clinical research and a completed CONSORT checklist must be included with all submissions.

Clinical trial registration: NCT02890641

Study protocol: Available on clinicaltrials.gov

Data collection: Patient recruitment was performed between 2016 and 2021 at the Fondation Ophtalmologique Adolphe de Rothschild in Paris, and consisted in the collection of surgical brain specimen and blood samples from patients who underwent focal drug-resistant epilepsy surgery. Data collection and analyses were performed between 2017 and 2024.

Outcomes: Not applicable, this clinical trial aims to collect samples.

Plants

Seed stocks

No plants were used

Novel plant genotypes

No plants were used

Authentication

No plants were used

Flow Cytometry

Plots

Confirm that:

☒ The axis labels state the marker and fluorochrome used (e.g. CD4-FITC).

☒ The axis scales are clearly visible. Include numbers along axes only for bottom left plot of group (a 'group' is an analysis of identical markers).

☒ All plots are contour plots with outliers or pseudocolor plots.

☐ A numerical value for number of cells or percentage (with statistics) is provided.

Methodology

Sample preparation

Nuclei were isolated from ~100mg of unfixed frozen cortical tissue from n = 7 FCDII cases. Isolation of nuclei was performed via dounce tissue homogenization, nuclei fixation in 1% PFA and sucrose gradient. Isolated nuclei were resuspended in staining buffer (2% BSA, 1mM EDTA, PBS), and immunostained overnight at 4°C with primary antibodies. The following day, nuclei were incubated 1h at 4°C with DAPI and fluorescent secondary antibodies for unconjugated OLIG2 and TBR1 primary antibodies.

Instrument

Moflo Astrios EQ (Beckman Coulter)

Software

Summit 6.3.1

Cell population abundance

pt11: PAX6+ / NEUN-, n=14,039 nuclei; OLIG2+ / NEUN-, n=63,398 nuclei; PU.1+ / NEUN-, n=7,142 nuclei; NEUN+, n=18,462 nuclei
pt12: PAX6+ / NEUN-, n=6,880 nuclei; OLIG2+ / NEUN-, n=24,603 nuclei; PU.1+ / NEUN-, n=5,537 nuclei; NEUN+, n=43,493 nuclei
pt4: PAX6+ / NEUN-, n=5,471 nuclei; OLIG2+ / NEUN-, n=1,578 nuclei; PU.1+ / NEUN-, n=1,917 nuclei; NEUN+, n=9,264 nuclei
pt13: PAX6+ / NEUN-, n=2,644 nuclei; OLIG2+ / NEUN-, n=40,648 nuclei; PU.1+ / NEUN-, n=5,512 nuclei; NEUN+, n=22,980 nuclei
pt14: PAX6+ / NEUN-, n=6,283 nuclei; OLIG2+ / NEUN-, n=30,043 nuclei; PU.1+ / NEUN-, n=32,197 nuclei; NEUN+, n=25,061 nuclei
pt15: PAX6+ / NEUN-, n=9,874 nuclei; OLIG2+ / NEUN-, n=32,350 nuclei; PU.1+ / NEUN-, n=25,619 nuclei; NEUN+, n=51,577 nuclei
pt9: PAX6+ / NEUN-, n=586 nuclei; OLIG2+ / NEUN-, n=7,117 nuclei; PU.1+ / NEUN-, n=18,182 nuclei; NEUN+/TBR1+, n=1,370 nuclei; NEUN+/TBR1-, n=23 nuclei

Gating strategy

Gating was performed following the steps listed below:
1. log SSC-A vs log DAPI-A
2. log SSC-A vs log FSC-A
3. FSC-H vs FSC-A
4. FSC-W vs FSC-A
5. log AF647-A vs log PE-A
6. log AF647-A vs log TBR1-A (only for samples stained for TBR1 from pt9)

☒ Tick this box to confirm that a figure exemplifying the gating strategy is provided in the Supplementary Information.
